# Side‐Chain Asymmetry in Quinoxaline Copolymer Donors Enables Optimal Morphology and Improved Charge Dynamics for High‐Efficiency Organic Solar Cells

**DOI:** 10.1002/advs.77555

**Published:** 2026-09-06

**Authors:** Can Zhu, Jinyuan Zhang, Ke Hu, Shirong Lu, Jianqi Zhang, Shucheng Qin, Hong Zhang, Yule Cheng, Wenbin Lai, Beibei Qiu, Jing Guo, Lei Meng, Gang Li, Yongfang Li

**Affiliations:** ^1^ Key Laboratory For Island Green Energy and New Materials School of Materials Science and Engineering Taizhou University Taizhou People's Republic of China; ^2^ Department of Electrical and Electronic Engineering Research Institute for Smart Energy (RISE) Photonic Research Institute (PRI) The Hong Kong Polytechnic University Hong Kong People's Republic of China; ^3^ Beijing National Laboratory for Molecular Sciences CAS Key Laboratory of Organic Solids Institute of Chemistry Chinese Academy of Sciences Beijing People's Republic of China; ^4^ CAS Key Laboratory of Nanosystem and Hierarchical Fabrication National Center for Nanoscience and Technology Beijing People's Republic of China; ^5^ Key Laboratory of Solid State Optoelectronic Devices of Zhejiang Province College of Physics and Electronic Information Engineering Zhejiang Normal University Jinhua Zhejiang People's Republic of China; ^6^ Province‐Ministry Co‐Construction Collaborative Innovation Center of Hebei Photovoltaic Technology Hebei Key Laboratory of Optic‐electronic Information and Materials College of Physics Science and Technology Hebei University Baoding Hebei People's Republic of China

**Keywords:** all‐polymer solar cells, balanced donor‐acceptor miscibility, quinoxaline‐based d‐a copolymer donors, side chain engineering

## Abstract

All‐polymer solar cells (all‐PSCs) have attracted increasing attention owing to their superior mechanical robustness, morphological stability, and solution‐processability. However, intricate intermolecular interactions hinder the precise regulation of donor‐acceptor miscibility and phase separation, thereby restricting the formation of ideal donor‐acceptor interpenetrating networks and favorable charge dynamics. Herein, an asymmetric side‐chain strategy is developed for a quinoxaline‐based polymer donor (PBAQ6) to precisely modulate donor and acceptor miscibility. Systematic studies demonstrate that the side‐chain structure could effectively modulate the film morphology features and charge dynamics of the active layer. Notably, the PBAQ6 polymer with an asymmetric side‐chain configuration achieves reasonable donor‐acceptor miscibility with the PYF‐T‐*o* polymer acceptor. Consequently, the PBAQ6:PYF‐T‐*o*‐based binary all‐PSCs deliver a power conversion efficiency (PCE) of 18.35%, and a further enhanced PCE of 19.52% is achieved for the ternary all‐PSCs with a small amount of PBQ12 as a second polymer donor, which ranks among the best values reported so far. Overall, this work demonstrates that, within the investigated quinoxaline‐based polymer‐donor platform, asymmetric side‐chain engineering can concurrently regulate donor–acceptor miscibility, active‐layer morphology, and bulk charge dynamics, thereby providing a useful molecular‐design principle for the development of high‐performance all‐polymer solar cells.

## Graphical abstract

Side‐chain asymmetry in quinoxaline copolymer donors modulates donor–acceptor miscibility and blend morphology in all‐polymer solar cells. The asymmetric donor PBAQ6 enables more balanced charge transport, reduced recombination, and improved charge dynamics with PYF‐T‐*o*, delivering PCEs of 18.35% in binary devices and 19.52% in ternary devices.

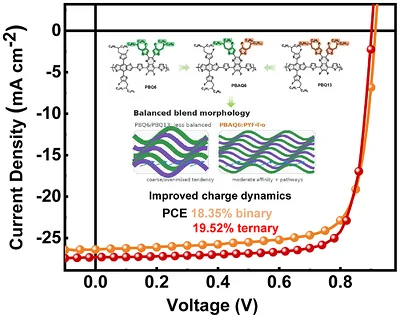

## 1 Introduction

Organic solar cells (OSCs) have been widely regarded as promising next‐generation photovoltaic technologies owing to their low weight, mechanical flexibility, and compatibility with low‐cost roll‐to‐roll manufacturing [1, 2, 3, 4, 5]. Over the past decade, the rapid development of narrow bandgap small molecule acceptors (SMAs), together with finely engineered donor polymers, has pushed the power conversion efficiencies (PCEs) of OSCs to above 20%, significantly narrowing the performance gap with inorganic photovoltaics [6, 7, 8, 9, 10, 11, 12]. Alongside this progress, all‐polymer solar cells (all‐PSCs) that integrate polymeric donors and polymeric acceptors are attracting growing interest, not only for their inherently enhanced mechanical robustness but also for their improved stability (morphological and operational) and broad synthetic tunability, which collectively favor large‐area processing and long‐term reliability [13, 14, 15].

Despite rapid progress enabled by the strategy of polymerized small‐molecule acceptors (PSMAs) [16, 17, 18], the efficiencies of all‐PSCs still generally lag behind the state‐of‐the‐art polymer:SMA‐based OSCs. A central origin of this discrepancy lies in the intrinsically more complex structure–property relationships in the all‐polymer blends. Compared with polymer:SMA systems, long conjugated backbones of polymer acceptors introduce stronger chain entanglement, reduced entropy of mixing, and more sluggish microstructure evolution, all of which complicate simultaneous optimization of crystallinity, nanoscale phase separation, and donor‐acceptor miscibility in the polymer donor‐polymer acceptor blends [19, 20, 21, 22]. Importantly, donor‐acceptor miscibility in all‐polymer blends should be neither too weak nor too strong. If miscibility is insufficient (i.e., a relatively large effective interaction parameter), coarse phase separation tends to occur, leading to reduced interfacial area for exciton dissociation and increased spatial heterogeneity that can aggravate trap formation and recombination. Conversely, excessively strong miscibility (i.e., an extremely small interaction parameter) may drive over‐mixing and compromise phase purity and percolation, disrupt continuous transport pathways, and increase transport‐related losses [23, 24]. Therefore, achieving an appropriate miscibility regime—where refined yet continuous phase separation coexists with sufficient packing and percolation—is crucial for concurrently maximizing charge generation and charge transport, and ultimately for closing the efficiency gap in the all‐PSCs [19, 20, 21, 22].

To address these issues, two complementary strategies have been widely explored. On the device‐engineering side, introducing a third component, optimizing solvent and additive systems, tailoring thermal or solvent‐annealing protocols, and employing temperature‐assisted coating have been used to finely tune the nanoscale phase separation [25, 26, 27]. From the molecular‐design perspective, extensive efforts have focused on engineering polymer acceptors through core modification, end‐group and π‐bridge engineering, and steric and hindrance control to suppress excessive chain entanglement and regulate aggregation (e.g., D18‐ChCl, PBDTSi‐*tt*TPD) [19, 20, 21, 28]. In parallel, side‐chain engineering on donor polymers has emerged as a particularly powerful handle to tune solubility, aggregation strength, and interchain packing, thereby influencing donor‐acceptor miscibility and phase behavior [21, 29, 30]. Collectively, these studies highlight that balanced miscibility and controlled nanoscale phase separation can reduce trap densities and suppress non‐radiative recombination, leading to higher fill factors (*FF*s) and improved operational stability in all‐PSCs [21, 29, 30]. Yet, a molecularly programmable strategy that deliberately tunes a miscibility regime—rather than simply “increasing miscibility”—remains highly desirable, especially for donor platforms that are scalable and versatile.

Quinoxaline‐based polymer donors are particularly attractive in this context because the quinoxaline unit provides accessible substitution sites along the conjugated backbone, enabling systematic side‐chain engineering without altering the underlying D‐A copolymer framework [31]. Such side‐chain modulation offers a multidimensional handle to simultaneously regulate solubility, frontier energy levels, aggregation behavior, and molecular packing, thereby governing donor‐acceptor miscibility and phase organization in all‐polymer blends [32, 33]. Representative quinoxaline‐based donors have demonstrated that rational substitution can support efficient charge separation even under minimal energetic offsets, as exemplified by PTQ11 [34, 35], while further side‐chain engineering and processing optimization have improved compatibility with polymer acceptors and boosted device efficiencies in all‐PSCs, as demonstrated by PBQ6 and PBQ8 [15, 36]. Nevertheless, robust morphology control remains challenging, highlighting the need for a molecularly programmable strategy that deliberately tunes donor‐acceptor interactions into an appropriate miscibility regime—achieving refined phase separation while maintaining percolated pathways and sufficient molecular order.

**FIGURE 1:**
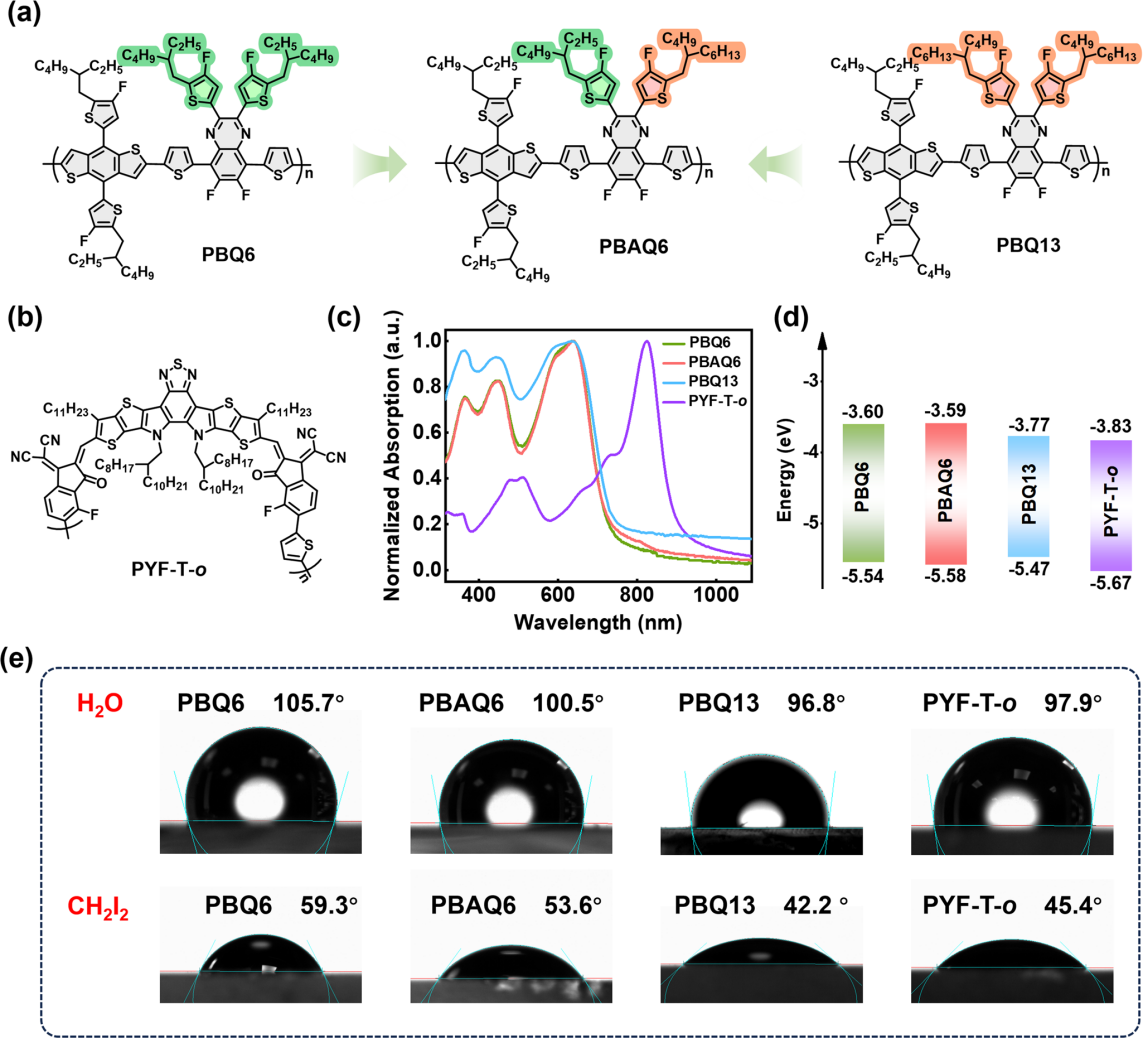
(a,b) Chemical structures of PBQ6, PBAQ6, PBQ13, and PYF‐T‐*o*, highlighting the side‐chain variations. (c) Normalized UV–vis absorption spectra of the neat polymer films. (d) Comparison of the frontier energy levels (HOMO and LUMO) of the polymers. (e) Contact‐angle measurements of water (H_2_O) and diiodomethane (CH_2_I_2_) on the pristine polymer films.

Here, we implement this concept on a quinoxaline‐based D‐A copolymer donor platform by systematically tailoring the length and branching of alkyl substituents on the quinoxaline acceptor unit. Two fluorothiophene‐based side‐chain motifs were introduced onto the quinoxaline (A) unit and copolymerized with a fluorinated benzo[1,2‐b:4,5‐b′]dithiophene (BDT‐F) donor (D) unit, yielding a donor series that enables direct evaluation of side‐chain architecture–miscibility–morphology relationships. Specifically, PBAQ6 incorporates two different alkyl‐substituted fluorothiophene motifs, whereas PBQ6 and PBQ13 serve as the corresponding symmetric‐side‐chain references. (Figure 1a). Unlike conventional side‐chain length or branching engineering, which mainly tunes solubility and aggregation through changes in side‐chain size or steric bulk, the present side‐chain‐asymmetry strategy combines two different alkyl‐substituted fluorothiophene motifs within the same quinoxaline‐based D–A copolymer framework to more subtly regulate donor–acceptor affinity, miscibility tendency, and blend morphology while preserving the conjugated backbone. To quantify donor–acceptor interactions, the PYF‐T‐*o* as polymer acceptor, static contact‐angle measurements were used to extract surface energies and estimate a relative interaction parameter (relative χ). Together with morphology investigations, these analyses establish that PBAQ6 resides in an intermediate relative χ regime, yielding refined yet continuous phase separation with a well‐percolated interpenetrating network while retaining a face‐on‐dominated packing motif with moderated coherence. Femtosecond transient absorption and transient optoelectronic characterizations further link this morphology–packing balance to device physics, revealing enhanced long‐lived charge signatures and slower charge decay, consistent with efficient charge generation and suppressed non‐productive recombination. Consequently, binary PBAQ6:PYF‐T‐*o* devices processed from non‐halogenated solvents deliver a PCE of 18.35% with a high fill factor (*FF*) of 76.12%, a short‐circuit current density (*J*_sc_) of 26.37 mA cm^−^^2^, and an open‐circuit voltage (*V*_oc_) of 0.914 V, while a ternary strategy with a small amount of second polymer donor PBQ12 [37] further boosts the efficiency to 19.52%. Overall, together with previous studies of asymmetric molecular design in diverse OSC materials [38, 39, 40], our results identify asymmetric side‐chain engineering as a practical, yet system‐dependent, molecular handle for regulating donor–acceptor miscibility, blend morphology, and charge dynamics in all‐PSCs.

## 2 Results and Discussion

Figure 1a,b depicts the molecular structures of the D–A copolymers PBQ6, PBAQ6, PBQ13 and the polymer acceptor PYF‐T‐*o*, respectively. To enable the synthesis of these new polymer donors, the corresponding monomers featuring tailored side‐chain architectures were prepared following the synthetic route outlined in Schemes S1–S3. Subsequent Pd‐catalyzed Stille coupling polymerization in sealed pressure tubes afforded the target copolymers. Detailed synthetic procedures, including reactant amounts, molar equivalents, reaction conditions, purification procedures, and isolated yields, together with full characterization data including ^1^H NMR, ^13^C NMR, ^19^F NMR, and mass spectrometry results for the new monomers and key intermediates, are provided in the Supporting Information, as shown in Figures S1–S12. All three copolymers exhibit good solubility in common halogenated solvents (e.g., chloroform, chlorobenzene, and o‐dichlorobenzene), and they are also readily soluble in non‐halogenated aromatic solvents such as toluene, o‐xylene, and 1,2,4‐trimethylbenzene. High‐temperature gel permeation chromatography (GPC) measurements reveal number‐average molecular weights (*M*_n_) of 22.6, 33.4, 13.8, and 17.1 kDa for PBQ6, PBAQ6, PBQ13, and PYF‐T‐*o*, respectively, with corresponding polydispersity indices (PDI) of 1.50, 2.32, 2.18, and 1.29. These values indicate reasonably narrow molecular‐weight distributions, and the detailed parameters are summarized in Figure S13.

As shown in the UV–vis absorption spectra and absorption‐coefficient spectra in Figure 1c and Figure S14, although PBQ6, PBAQ6, and PBQ13 differ in their side chains, they share an identical conjugated backbone and therefore exhibit very similar absorption characteristics in both solution and solid film. In solution, the three polymer donors display main absorption maxima at 617, 618, and 623 nm, respectively. Upon film formation, these bands red‐shift by ca. 12–20 nm to 637, 638, and 635 nm, respectively, indicating strengthened interchain interactions in the condensed phase. The absorption onsets of the neat films extend to 720, 713, and 732 nm, corresponding to optical bandgaps of 1.72, 1.74, and 1.69 eV for PBQ6, PBAQ6, and PBQ13, respectively. The polymer acceptor PYF‐T‐*o* exhibits a main absorption peak at 811 nm in solution, which red‐shifts to 825 nm in the film with an absorption edge around 888 nm, yielding an optical bandgap of 1.40 eV. These results clearly demonstrate that the three donors provide highly complementary absorption with the polymer acceptor PYF‐T‐*o* covering the 400–900 nm region, which is beneficial for extending the coverage of the visible–near‐infrared solar spectrum and thus for achieving higher short‐circuit current densities (*J*_sc_). As summarized in Figure S14b, the maximum absorption coefficients of the PBQ6, PBAQ6, and PBQ13 neat films reach 7.24 ×  10^4^, 7.58 ×  10^4^, and 6.55 ×  10^4^ cm^−1^, respectively, with PBAQ6 exhibiting a slightly larger coefficient than the other two donors. The PBQ6:PYF‐T‐*o*, PBAQ6:PYF‐T‐*o*, and PBQ13:PYF‐T‐*o* blend films show nearly identical absorption from 400 to 900 nm (Figure S14c), with maximum absorption coefficients of 5.62 ×  10^4^, 5.84 ×  10^4^, and 5.15  ×  10^4^ cm^−1^, respectively (Figure S14d). The higher absorption coefficients of the PBAQ6‐based blend films should lead to larger *J*_sc_ in the corresponding devices, all else being equal.

The onset oxidation and reduction potentials of PBQ6, PBAQ6, PBQ13, and the polymer acceptor PYF‐T‐*o* were determined by cyclic voltammetry (CV) in Figures S15 and S16. Using Ag/AgCl as the reference electrode and the Fc/Fc^+^ redox couple (0.45 V vs. Ag/AgCl) as an internal standard (experimental details are provided in the Supporting Information), the frontier orbital energy levels (HOMO and LUMO) were converted to values relative to the vacuum level according to *E*_HOMO_/*E*_LUMO_ = −e (*φ*_ox_/*φ*_red_ + 4.8 – *φ*_Fc/Fc+_) (eV). On this basis, the *E*_HOMO_ and *E*_LUMO_ values of PBQ6, PBAQ6, and PBQ13 are evaluated to be −5.54 and −3.60, −5.58 and −3.59, and −5.47 and −3.77 eV, respectively, while PYF‐T‐*o* exhibits *E*_HOMO_ and *E*_LUMO_ of −5.67 and −3.83 eV (Figure 1d). As shown in the density functional theory (DFT) calculated frontier molecular orbital distributions based on four‐repeating‐unit oligomer models in Figure S17, both the HOMO and LUMO of PBQ6, PBAQ6, and PBQ13 are mainly delocalized along the conjugated backbones, indicating that the three polymers retain similar backbone electronic structures despite their different side‐chain architectures. This result indicates that side‐chain asymmetry does not substantially alter the frontier orbital distribution of the conjugated backbone, but may primarily affect donor‐acceptor miscibility, molecular packing, and blend morphology.

Increasing the side‐chain bulk (e.g., by introducing longer alkyl substituents) and implementing side‐chain asymmetry generally enhances the solubility of conjugated polymers in processing solvents and weakens excessive interchain aggregation, thereby providing a structural basis for improved donor‐acceptor compatibility [19, 41]. To quantitatively evaluate the interfacial compatibility between the different polymer donors and the polymer acceptor PYF‐T‐*o*, static contact‐angle measurements were performed using water and diiodomethane as probe liquids to determine the surface energy (surface tension, ***γ***) of the neat films (Figure 1e and Table S1). The surface energies were calculated using the Owens–Wendt–Rabel–Kaelble (OWRK) method, in which the total surface energy is divided into dispersive and polar components [42]. PYF‐T‐*o* exhibits a ***γ*** of 40.2 mN m^−1^, whereas PBQ6, PBAQ6, and PBQ13 show ***γ*** values of 29.3, 33.0, and 42.8 mN m^−1^, respectively, indicating that side‐chain modulation changes the surface‐energy matching between the donor and acceptor. Furthermore, the donor‐acceptor miscibility was assessed using the Flory–Huggins interaction parameter, which can be estimated by the empirical relation:

$$\chi =K{\left(\sqrt{\gamma_{d}}-\sqrt{\gamma_{a}}\right)}^{2}$$

where *K* is a constant and ***γ*** *_d_* and ***γ*** *_a_* denote the surface energies of the donor and acceptor, respectively. For comparison, we report the relative parameter relative χ. While a smaller relative χ generally indicates stronger donor‐acceptor affinity, excessively small relative χ may lead to over‐miscibility, which can reduce the purity of percolated donor‐acceptor domains and compromise crystallinity and charge‐transport pathways. Therefore, an intermediate relative χ is often desirable to balance sufficient interfacial area for exciton dissociation with adequate phase purity for efficient carrier transport. Accordingly, the relative χ values for PBQ6:PYF‐T‐*o*, PBAQ6:PYF‐T‐*o*, and PBQ13:PYF‐T‐*o* were determined to be 0.86, 0.35, and 0.04 *K*, respectively. Despite having similar molecular weights, PBAQ6 and PBQ13 showed significantly divergent relative χ values, measuring 0.35 *K* and 0.03 *K*, respectively (Table S1). The larger relative χ of PBQ6 suggests weaker donor–acceptor affinity, whereas the very small relative χ of PBQ13 suggests stronger apparent miscibility. In comparison, PBAQ6 exhibits an intermediate relative χ, indicating a more moderate donor–acceptor affinity. The detailed morphology–performance relationship is further evaluated below by combining atomic force microscopy (AFM), grazing‐incidence wide‐angle x‐ray scattering (GIWAXS), charge‐transport, recombination, and photovoltaic measurements.

**FIGURE 2:**
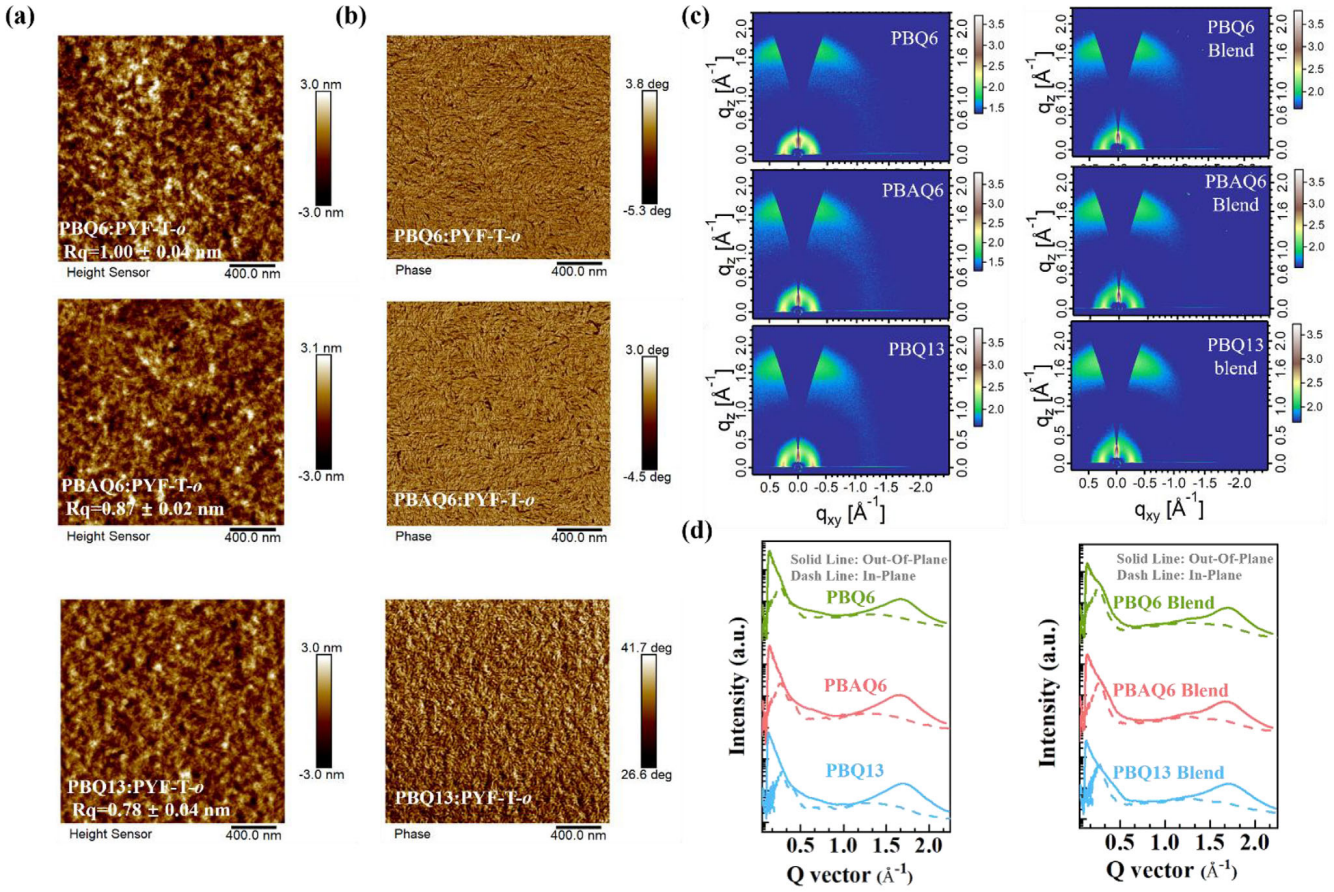
(a) AFM height (topography) images and (b) corresponding phase images of the PBQ6:PYF‐T‐*o*, PBAQ6:PYF‐T‐*o*, and PBQ13:PYF‐T‐*o* blend films (*R*_q_ values are shown in the panels; scale bars: 400 nm). (c) 2D GIWAXS patterns of the neat PBQ6, PBAQ6, and PBQ13 films and the corresponding 1D line cuts extracted along the out‐of‐plane (solid lines) and in‐plane (dashed lines) directions. (d) 2D GIWAXS patterns and the corresponding out‐of‐plane and in‐plane line cuts of the PBQ6:PYF‐T‐*o*, PBAQ6:PYF‐T‐*o*, and PBQ13:PYF‐T‐*o* blend films.

To examine the influence of donor–acceptor miscibility on the active‐layer morphology, the blend films were characterized by AFM and transmission electron microscopy (TEM). As shown in Figure 2a,b and Table S2, all blend films exhibit continuous and compact surfaces without obvious pinholes or micron‐scale aggregates, indicating good film‐forming properties under the optimized processing conditions. Repeated AFM measurements at three different locations yield average *R*_q_ values of 1.00 ± 0.04, 0.87 ± 0.02, and 0.78 ± 0.04 nm for the PBQ6, PBAQ6, and PBQ13 blend films, respectively. These results indicate that all three blend films possess relatively smooth surfaces, with modest but reproducible variations in surface roughness. The PBQ6 blend exhibits the highest average *R*_q_ and a somewhat more heterogeneous phase texture, consistent with its relatively larger χ value and a greater tendency toward nanoscale phase separation. In comparison, the PBAQ6 blend shows an intermediate *R*_q_ together with a comparatively uniform phase distribution, suggesting a more balanced donor–acceptor miscibility and nanoscale phase organization. The PBQ13 blend exhibits the lowest average *R*_q_ and a fine‐scale phase texture, consistent with its smaller relative χ value and relatively stronger miscibility. Despite their comparable molecular weights, PBAQ6:PYF‐T‐*o* and PBQ13:PYF‐T‐*o* exhibited similarly smooth surfaces, with *R*_q_ values of 0.87 and 0.82 nm (Figure S18), respectively. The TEM images and quantitative grayscale analysis of the corresponding blend films are shown in Figure S19 and Table S3, respectively. The TEM images display broadly similar contrast among the three blend films, and the differences are relatively subtle. To further compare the image uniformity and local intensity fluctuations, quantitative grayscale analysis was performed. The PBQ6 blend shows a slightly higher grayscale standard deviation and normalized RMS contrast of 29.3 and 21.43%, respectively, compared with PBAQ6 (27.0% and 19.30%) and PBQ13 (26.0% and 19.09%). These results indicate that PBQ6 exhibits slightly stronger local intensity fluctuations, whereas PBAQ6 and PBQ13 show comparatively more uniform grayscale contrast at this length scale. Together with the AFM results, the slightly higher *R*_q_ and TEM contrast fluctuation of PBQ6 suggest somewhat stronger local morphological heterogeneity.

To probe molecular packing and preferential orientation, GIWAXS measurements were performed on neat films of PBQ6, PBAQ6, PBQ13, and PYF‐T‐*o* (Figure 2c and the corresponding 1D line cuts). All three donor films exhibit a pronounced face‐on preference, as evidenced by the (010) π‐π stacking peaks predominantly located in the out‐of‐plane (OOP) direction at *q* ≈1.704, 1.684, and 1.686 Å^−1^, corresponding to *d*_π‐π_ values of ca. 3.690, 3.731, and 3.727 Å, respectively (Table S4). The extracted coherence lengths (CCL) are 25.11, 22.58, and 19.52 Å, indicating distinct degrees of crystallinity order among the donors. In contrast, PYF‐T‐*o* shows a comparatively weaker (010) signal in the OOP direction (*q* ≈ 1.599 Å^−1^, *d*_π‐π_≈3.93 Å; in Figure S20), while displaying a more pronounced (100) lamellar peak in the in‐plane (IP) direction (*q* ≈ 0.413 Å^−1^, *d*_100_≈15.20 Å), suggesting a less pronounced face‐on preference compared with the donor polymers. Notably, relative to PBQ6, PBAQ6 exhibits a slightly enlarged *d*_π‐π_ and a reduced CCL, implying modestly relaxed π‐π packing and decreased ordering, which can be attributed to the side‐chain asymmetry‐induced weakening of interchain aggregation.

To further investigate the molecular orientation and packing evolution in the blend films, GIWAXS measurements were conducted (Figure 2d and Table S4). All blends retain a predominantly face‐on texture, evidenced by the (010) π–π stacking diffraction mainly distributed along the OOP direction. The PBQ6:PYF‐T‐*o* blend shows a clear (010) peak at *q* ≈ 1.672 Å^−1^ (*d*_π‐π_≈3.759 Å). In comparison, PBAQ6:PYF‐T‐*o* blend exhibits a down‐shifted (010) feature at *q* ≈ 1.631 Å^−1^ (*d*_π‐π_≈3.853 Å), whereas the (010) signal of PBQ13:PYF‐T‐*o* is overall weaker and more diffuse, with a peak at *q* ≈ 1.675 Å^−1^ (*d*_π‐π_≈3.751 Å). Importantly, these GIWAXS signatures are consistent with the miscibility–morphology trends inferred from the surface‐energy‐derived relative interaction parameter relative χ and morphology investigations. PBQ6:PYF‐T‐*o*, with the largest relative χ, also shows coarser phase separation, as evidenced by higher surface roughness and more pronounced phase contrast, while retaining relatively preserved packing coherence, reflected by a comparatively larger (010) π–π coherence length (CCL = 21.51 Å). At the opposite extreme, PBQ13:PYF‐T‐*o* (extremely small relative χ) yields smooth surfaces yet pronounced fine‐scale heterogeneity, accompanied by a weakened (010) coherence (CCL = 17.92 Å) but markedly enhanced lamellar ordering (CCL_100_ = 94.49 Å; *q*_100_ ≈0.268 Å^−1^, *d*_100_≈23.434 Å), indicative of lamella‐dominated ordering and domain purification that may be less favorable for continuous π–π‐mediated transport. Notably, PBAQ6:PYF‐T‐*o* shows an intermediate contact‐angle‐derived relative χ and exhibits comparatively balanced structural features in the blend film. Correspondingly, its (010) peak is down‐shifted and broadened (*q* ≈ 1.631 Å^−1^, *d*_π‐π_≈3.853 Å; FWHM = 0.318 Å^−1^; CCL = 17.97 Å), while maintaining a well‐defined lamellar framework (*q*_100_ ≈ 0.264 Å^−1^, *d*_100_≈23.811 Å; CCL_100_ = 58.01 Å). This combination points to moderately relaxed π–π packing coupled with preserved mesoscale order, enabling refined phase separation without sacrificing the short‐range order and percolation required for efficient charge transport.

**TABLE 1:** Molecular weight and physicochemical properties of PBQ6, PBAQ6, PBQ13, and PYF‐T‐*o*.

Polymer materials	*M*_n_ (kDa)	PDI	λ_max_ film (nm)	λ_onset_ film (nm)	$E_{g}^{opt}$(eV)^a^	*E*_HOMO_ (eV)^b^	*E*_LUMO_ (eV)^b^	*µ*_h_ (10^−4^ cm^2^V^−1^s^−1^)
PBQ6	22.6	1.50	637	720	1.72	−5.54	−3.60	2.41
PBAQ6	33.4	2.32	638	713	1.74	−5.58	−3.59	2.30
PBQ13	13.8	2.18	635	732	1.69	−5.47	−3.77	2.09
PYF‐T‐*o*	17.1	1.29	825	888	1.40	−5.67	−3.83	∖

^a^ Calculated from the absorption edge of the polymer films: $E_{g}^{opt}$= 1240/ *λ*_onset_

^b^ Calculated by cyclic voltammetry (CV).

**TABLE 2:** Photovoltaic parameters of the OSCs based on polymer donors: PYF‐T‐*o* under illumination of AM 1.5G (100 mW cm^−2^). The donor‐acceptor ratio is 1:1.1, and the active layer thickness is 110 nm.

Blend films	*V*_oc_ (V)	*J*_sc_ (mA cm^−2^)	*J*_cal_ ^c^ (mA cm^−2^)	*FF* (%)	PCE (%)
PBQ6:PYF‐T‐*o* ^b^	0.890 (0.882 ± 0.009)	24.72 (24.33 ± 0.39)	24.12 (23.85 ± 0.27)	73.21 (72.34 ± 0.87)	16.11 (15.93 ± 0.19)^a^
PBAQ6:PYF‐T‐*o* ^b^	0.910 (0.902 ± 0.008)	25.02 (24.75 ± 0.27)	24.32 (24.06 ± 0.26)	77.01 (76.31 ± 0.70)	17.53 (17.18 ± 0.35)
PBQ13:PYF‐T‐*o* ^b^	0.922 (0.914 ± 0.008)	24.27 (23.85 ± 0.42)	22.98 (22.45 ± 0.53)	66.17 (65.31 ± 0.86)	14.81 (14.27 ± 0.53)
PBAQ6:PYF‐T‐*o* ^d^	0.914 (0.903 ± 0.011)	26.37 (25.95 ± 0.42)	25.78 (25.50 ± 0.28)	76.12 (75.51 ± 0.55)	18.35 (17.75 ± 0.60)
PBAQ6:PBQ12:PYF‐T‐*o* ^d^	0.904 (0.887 ± 0.017)	27.31 (26.93 ± 0.37)	26.53 (26.35 ± 0.18)	79.08 (78.39 ± 0.69)	19.52 (18.95 ± 0.57)

^a^ Average values and standard deviation obtained from more than ten different devices.

^b^ Fabricated with optimal conditions.

^c^ Integrated from EQE values.

^d^ The 2PACz as the HTL.

**FIGURE 3:**
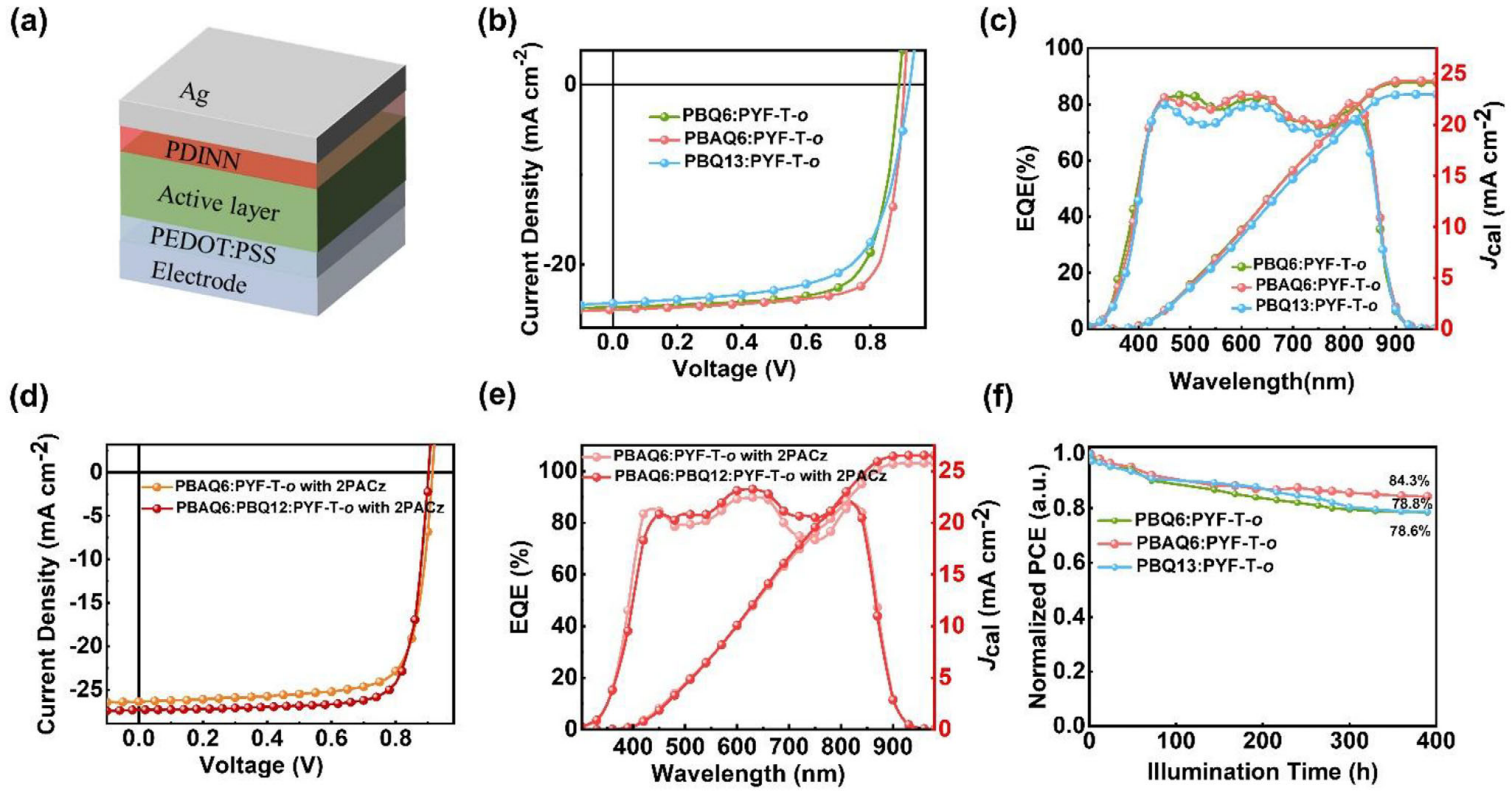
(a) Schematic device architecture of the all‐PSCs. (b) *J–V* curves of devices based on PBQ6:PYF‐T‐*o*, PBAQ6:PYF‐T‐*o*, and PBQ13:PYF‐T‐*o* active layers. (c) EQE curves and the corresponding *J*_cal_ of the three devices. (d) *J–V* characteristics of PBAQ6:PYF‐T‐*o* and PBAQ6:PBQ12:PYF‐T‐*o* devices employing 2PACz as the hole transport layer (HTL). (e) EQE curves and integrated *J*_cal_ of PBAQ6‐based devices with 2PACz as HTL. (f) Normalized PCE evolution of the three devices under continuous illumination, demonstrating operational stability.

To verify whether side‐chain asymmetry, through its regulation of donor‐acceptor miscibility and blend morphology, could translate into improved photovoltaic performance, we fabricated all‐PSCs using PBQ6, PBAQ6, or PBQ13 as the donor and PYF‐T‐*o* as the acceptor. A conventional device structure of ITO/PEDOT:PSS (or 2PACz)/active layer/PDINN/Ag was employed (Figure 3a). Because non‐halogenated processing is particularly sensitive to film‐formation kinetics and phase separation, toluene was selected as the casting solvent [43, 44]. We then systematically optimized key processing parameters—including the additive type and loading, the donor‐acceptor mass ratio, and the solution concentration (Tables S5–S7). The best performance was obtained with a donor‐acceptor mass ratio of 1:1.1, a total concentration of 18 mg mL^−1^, 1.2 vol% 1‐chloronaphthalene (CN) as the additive, followed by thermal annealing at 95°C for 5–10 min. In addition, given the relatively high boiling point of toluene (110.6°C), both the solution and the substrate were preheated to ∼60°C during spin‐coating. This mild heating accelerates solvent evaporation and helps avoid excessive phase separation, thereby yielding more stable and reproducible blend morphologies [45]. Figure 3b shows representative current density‐voltage (*J‐V*) characteristics measured under AM 1.5G illumination, and the corresponding photovoltaic parameters are summarized in Table 2. With symmetric side‐chain donors, PBQ6:PYF‐T‐*o* and PBQ13:PYF‐T‐*o* delivered PCEs of 16.11% and 14.81%, respectively, with *V*_oc_ values of 0.89 and 0.92 V, *J*_sc_ values of 24.72 and 24.27 mA cm^−2^, and *FF* of 73.21% and 66.17%. Under identical processing conditions, replacing the donor with the asymmetric‐side‐chain polymer PBAQ6 led to a clear performance improvement: the PCE increased to 17.53% with a higher *FF* of 77.01%, while maintaining a high *J*_sc_ (25.02 mA cm^−2^) and a comparable *V*_oc_ (0.91 V). Despite the similar molecular weights of PBAQ6 and PBQ13 (Figure S26 and Table S8), PBAQ6 still exhibits superior photovoltaic performance (Figure S21 and Table S9). Regarding the photocurrent generation, the variation in the *J*_sc_ follows the trend of the absorption coefficients (Figure S14). Notably, considering the relative interaction parameters (relative χ) derived from contact‐angle measurements together with the morphology investigation results, the improved *FF* is consistent with the PBAQ6:PYF‐T‐*o* active layer residing in an appropriate miscibility regime, which refines phase separation and strengthens the continuity of the interpenetrating network, ultimately reducing transport losses and non‐productive recombination [19]. Furthermore, replacing the hole‐transporting layer with 2PACz boosted the *J*_sc_ and enhanced the overall device performance. The PCE for the PBAQ6:PYF‐T‐*o* device was elevated to 18.35% (Figure 3d and Table 2). Introducing PBQ12 as a third component further optimized the device performance, yielding a maximum PCE of 19.52%. Owing to its higher‐lying HOMO level (‐5.40 eV) and relatively high charge‐carrier mobility, PBQ12 enables favorable stepwise energy‐level alignment with PBAQ6 and PYF‐T‐*o*, thereby facilitating hole transfer and charge extraction [37, 46]. Although this higher HOMO slightly reduces *V*_oc_ by narrowing the effective photovoltaic gap, the resulting gains in *J*_sc_ and *FF* compensate for this loss and lead to an overall PCE enhancement. In summary, the side‐chain‐asymmetry strategy is implemented on a common quinoxaline‐based D–A copolymer backbone through simple side‐chain variation, while retaining good solubility in non‐halogenated aromatic solvents such as toluene and o‐xylene. Such non‐halogenated solution processability is highly desirable for future large‐area fabrication of all‐polymer solar cells [47, 48]. The asymmetrically engineered PBAQ6‐based all‐PSCs demonstrate state‐of‐the‐art photovoltaic performance and highlight the effectiveness of side‐chain asymmetry in developing high‐performance and processable polymer donors.

To further corroborate the origin of the photocurrent and verify the reliability of the measured *J*_sc_, the external quantum efficiency (EQE) spectra of the champion devices were recorded (Figure 3c,e). All devices exhibit pronounced photo‐response across 400–850 nm, with EQE values generally exceeding 80% in the 400–650 nm region. This similarity is consistent with the closely related conjugated backbones of the three donors and their comparable absorption characteristics, leading to nearly identical spectral response. Importantly, the integrated current densities derived from the EQE spectra agree well with the values obtained from *J–V* measurements. With PEDOT:PSS as the hole‐transport layer, the calculated currents *J*_cal_ for the devices based on PBQ6:PYF‐T‐*o*, PBAQ6:PYF‐T‐*o*, and PBQ13:PYF‐T‐*o* are 24.12, 24.32, and 22.98 mA cm^−2^, respectively, following the same trend as *J*_sc_. This agreement rules out measurement artefacts and indicates that the enhanced device output is primarily associated with an increased generation and extraction of photocarriers. After replacing PEDOT:PSS with 2PACz, *J*_cal_ for the device based on PBAQ6:PYF‐T‐*o* rises to 25.78 mA cm^−2^; moreover, implementing and optimizing a ternary strategy further increases *J*_cal_ to 26.53 mA cm^−2^.

Beyond high efficiency, operational stability under continuous illumination is a critical figure of merit for all‐PSCs. Accordingly, we monitored the stability of optimized devices based on PBQ6:PYF‐T‐*o*, PBAQ6:PYF‐T‐*o*, and PBQ13:PYF‐T‐*o* under continuous illumination (Figure 3f). All the devices show a noticeable initial efficiency loss, followed by a slower decay at longer times. Among them, the device based on PBAQ6:PYF‐T‐*o* delivers superior long‐term stability, retaining 84.3% of its initial PCE after nearly 400 h of light soaking, outperforming the other two systems. This enhanced stability is consistent with the blend of PBAQ6:PYF‐T‐*o* residing in an appropriate miscibility regime, as indicated by the relative interaction parameter (relative χ) derived from contact‐angle measurements and corroborated by morphology investigations. The refined yet continuous interpenetrating network and the balanced molecular packing and phase organization are expected to suppress illumination‐induced morphological evolution, such as domain coarsening or aggravated phase separation, thereby sustaining charge‐transport percolation and mitigating performance degradation over extended operation.

To more clearly elucidate how side‐chain asymmetry in the polymer donor influences charge‐transport properties in the all‐PSCs, electron (*µ*_e_) and hole (*µ*_h_) mobilities were measured by the space‐charge‐limited current (SCLC) method for neat donor films and the corresponding blend films (Figure S22 and Table 1 and Table S10). In the neat donor films, the hole mobilities of all three polymers are on the order of 10^−4^ cm^2^V^−1^s^−1^ and differ only slightly, indicating that the side‐chain modification has a limited impact on the intrinsic hole‐transport capability of the donors. In contrast, pronounced differences emerge upon blending with the polymer acceptor of PYF‐T‐*o*. PBAQ6:PYF‐T‐*o* blend exhibits simultaneously high *µ*_e_ and *µ*_h_ of 7.63  ×  10^−4^ cm^2^V^−1^s^−1^and 6.80  ×  10^−4^ cm^2^V^−1^s^−1^, together with a more balanced transport (*µ*_e_/*µ*_h_ = 1.12). For the blend film of PBQ6:PYF‐T‐*o*, *µ*_e_ and *µ*_h_ are 7.50  ×  10^−4^ cm^2^V^−1^s^−1^ and 6.36  ×  10^−4^ cm^2^V^−1^s^−1^, corresponding to *µ*_e_/*µ*_h_ = 1.18. By comparison, the PBQ13:PYF‐T‐*o* blend shows markedly reduced mobilities (*µ*_e_ = 2.27  ×  10^−4^ cm^2^V^−1^s^−1^, *µ*_h_ = 1.68  ×  10^−4^ cm^2^V^−1^s^−1^) and a larger transport imbalance (*µ*_e_/*µ*_h_ = 1.35). Such balanced bipolar transport (i.e., *µ*_e_/*µ*_h_ closer to unity) is generally favorable for suppressing space‐charge accumulation and reducing transport‐related recombination, thereby supporting a higher *FF* [49, 50]. These transport trends are consistent with the miscibility and morphology analyses, suggesting that the donor–acceptor an appropriate miscibility regime and the more refined yet well‐connected interpenetrating network in PBAQ6:PYF‐T‐*o* enable more balanced bipolar transport and underpin its superior *FF*.

**FIGURE 4:**
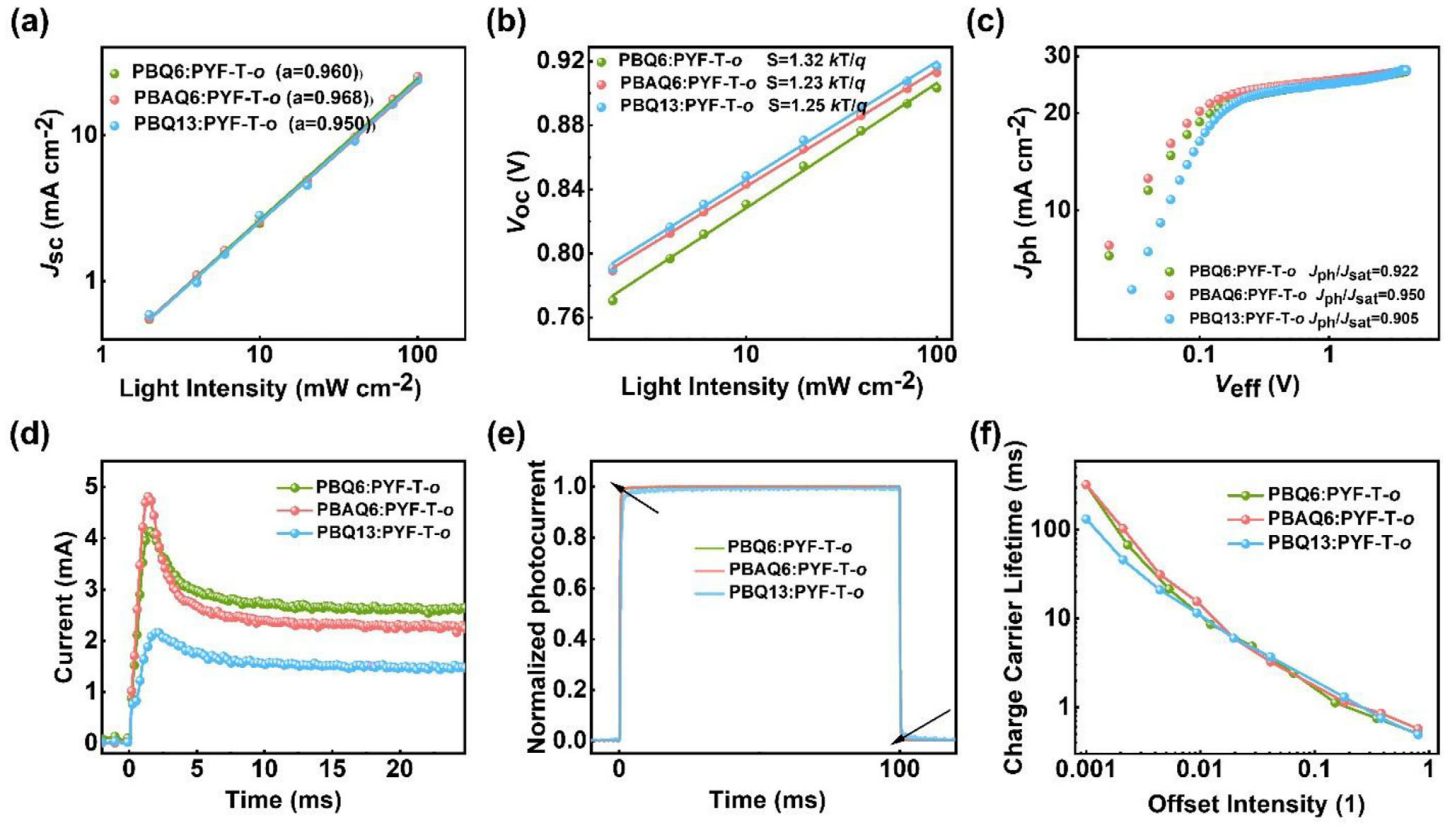
(a) Light‐intensity dependence of *J*_sc_ for PBQ6:PYF‐T‐*o*, PBAQ6:PYF‐T‐*o*, and PBQ13:PYF‐T‐*o* devices. (b) Light‐intensity dependence of Voc. (c) *J*_ph_ vs. *V*_eff_ of the all‐PSCs. (d) Photo‐CELIV curves of the three devices. (e) Corresponding TPC curves. (f) Carrier lifetime curves under different light intensities obtained from TPV measurements.

To elucidate the charge recombination dynamics and exciton dissociation probabilities within the active layer, we examined the light‐intensity (*P*_light_) dependence of *J*_sc_ and *V*_oc_, as well as the dependence of the photocurrent density (*J*_ph_) on the effective voltage (*V*_eff_) (Figure 4a–c). The *J*_sc_—*P*_light_ dependence follows a power‐law behavior, *J_sc_* ∝*P*_light_ *^α^*, where α is the exponent; an α value approaching unity typically indicates suppressed bimolecular recombination under short‐circuit conditions [15, 51]. As shown in Figure 4a, the slopes of log *J_sc_* vs. log *P*_light_ yield α values of 0.960, 0.968, and 0.950 for the PBQ6:PYF‐T‐*o*, PBAQ6:PYF‐T‐*o*, and PBQ13:PYF‐T‐*o* devices, respectively, suggesting that bimolecular recombination is generally weak in all three systems, with the PBAQ6‐based device exhibiting the most favorable trend. Then we evaluated the *V*_oc_‐ ln *P*_light_ dependence to probe trap‐assisted recombination. In this analysis, a slope closer to 1*kT*/*q* implies a reduced contribution from Shockley–Read–Hall (SRH) recombination [52]. The extracted slopes are 1.32*kT*/*q*, 1.23*kT*/*q*, and 1.25*kT*/*q* for the PBQ6:PYF‐T‐*o*, PBAQ6:PYF‐T‐*o*, and PBQ13:PYF‐T‐*o* devices, respectively, indicating that trap‐assisted recombination is more effectively suppressed in the device based on PBAQ6:PYF‐T‐*o*. Furthermore, the *J*_ph_‐*V*_eff_ characteristics were analyzed to assess how miscibility‐driven morphological differences affect exciton dissociation and charge collection [53, 54]. Here, *J*_ph_ = *J*_L_‐*J*_D_, where *J*_L_ and *J*_D_ are the current densities under illumination and in the dark, respectively. The *V*_eff_ is defined as *V*_eff_ = *V*_0_‐*V*, where *V*_0_ is the voltage at which *J*_ph_ = 0 and *V* is the applied bias. When *V*_eff_>2 V, *J*_ph_ reaches saturation (*J*_sat_), implying nearly complete field‐driven exciton dissociation and efficient carrier collection at the electrodes. Accordingly, the ratio *J*_ph_/*J*_sat_ can be used to estimate the combined probability of exciton dissociation and charge collection, denoted as *P*(E, T). The extracted *P*(E, T) values are 92.2%, 95.0%, and 90.5% for the devices based on PBQ6:PYF‐T‐*o*, PBAQ6:PYF‐T‐*o*, and PBQ13:PYF‐T‐*o*, respectively, demonstrating that the PBAQ6‐based device achieves the highest dissociation and collection probability. Taken together, these results indicate that the device based on PBAQ6:PYF‐T‐*o* exhibits reduced recombination losses and more efficient exciton dissociation and charge collection, which collectively underpin its simultaneously enhanced *FF*, *J*_sc_, and overall device efficiency in the all‐PSCs.

To gain deeper insight into charge transport and carrier recombination under operating illumination, we carried out photo‐induced charge extraction by linearly increasing voltage (photo‐CELIV), transient photocurrent (TPC), and transient photovoltage (TPV) measurements for the three all‐PSCs (Figure 4d–f). Compared with single‐carrier SCLC measurements, photo‐CELIV more directly captures the effective extraction kinetics and drift transport in working devices under illumination. As shown in Figure 4d, the PBAQ6:PYF‐T‐*o* device exhibits the highest photo‐CELIV‐derived carrier mobility of 3.1  ×  10^−4^ cm^2^V^−1^s^−1^, exceeding those of PBQ6:PYF‐T‐*o* (2.6  ×  10^−4^ cm^2^V^−1^s^−1^) and PBQ13:PYF‐T‐*o* (2.1  ×  10^−4^ cm^2^V^−1^s^−1^), in line with the higher *J*_sc_ observed for the PBAQ6‐based devices.

We further employed TPC to probe space‐charge effects as well as carrier trapping and de‐trapping processes in the all‐PSCs. Figure 4e shows the TPC traces of the devices based on PBQ6:PYF‐T‐*o*, PBAQ6:PYF‐T‐*o*, and PBQ13:PYF‐T‐*o*. The current rises promptly upon the light pulse (Figure S23a) and decays rapidly after the pulse is switched off (Figure S23b). Exponential fitting of the decay yields time constants of 0.192, 0.162, and 0.202 µs for the devices based on PBQ6:PYF‐T‐*o*, PBAQ6:PYF‐T‐*o*, and PBQ13:PYF‐T‐*o*, respectively. Notably, the shorter decay time for the PBAQ6‐based device suggests faster charge extraction and reduced carrier retention associated with trapping or space‐charge effects. In parallel, TPV measurements were performed to assess recombination dynamics under open‐circuit conditions (Figure 4f). With increasing bias light intensity, the TPV decay lifetime τ decreases monotonically for all devices, consistent with accelerated recombination at higher carrier densities. Importantly, the PBAQ6:PYF‐T‐*o* device maintains a comparatively longer τ across the entire intensity range; at the highest bias light, τ reaches 0.572 µs, longer than those of PBQ6:PYF‐T‐*o* (0.503 µs) and PBQ13:PYF‐T‐*o* (0.492 µs), indicative of a lower effective recombination rate. Taken together, these kinetic signatures are consistent with PBAQ6:PYF‐T‐*o* residing in an appropriate miscibility regime, where the refined yet continuous interpenetrating network facilitates efficient carrier transport and extraction while suppressing recombination losses in devices, thereby supporting the enhanced *J*_sc_ and *FF*.

**FIGURE 5:**
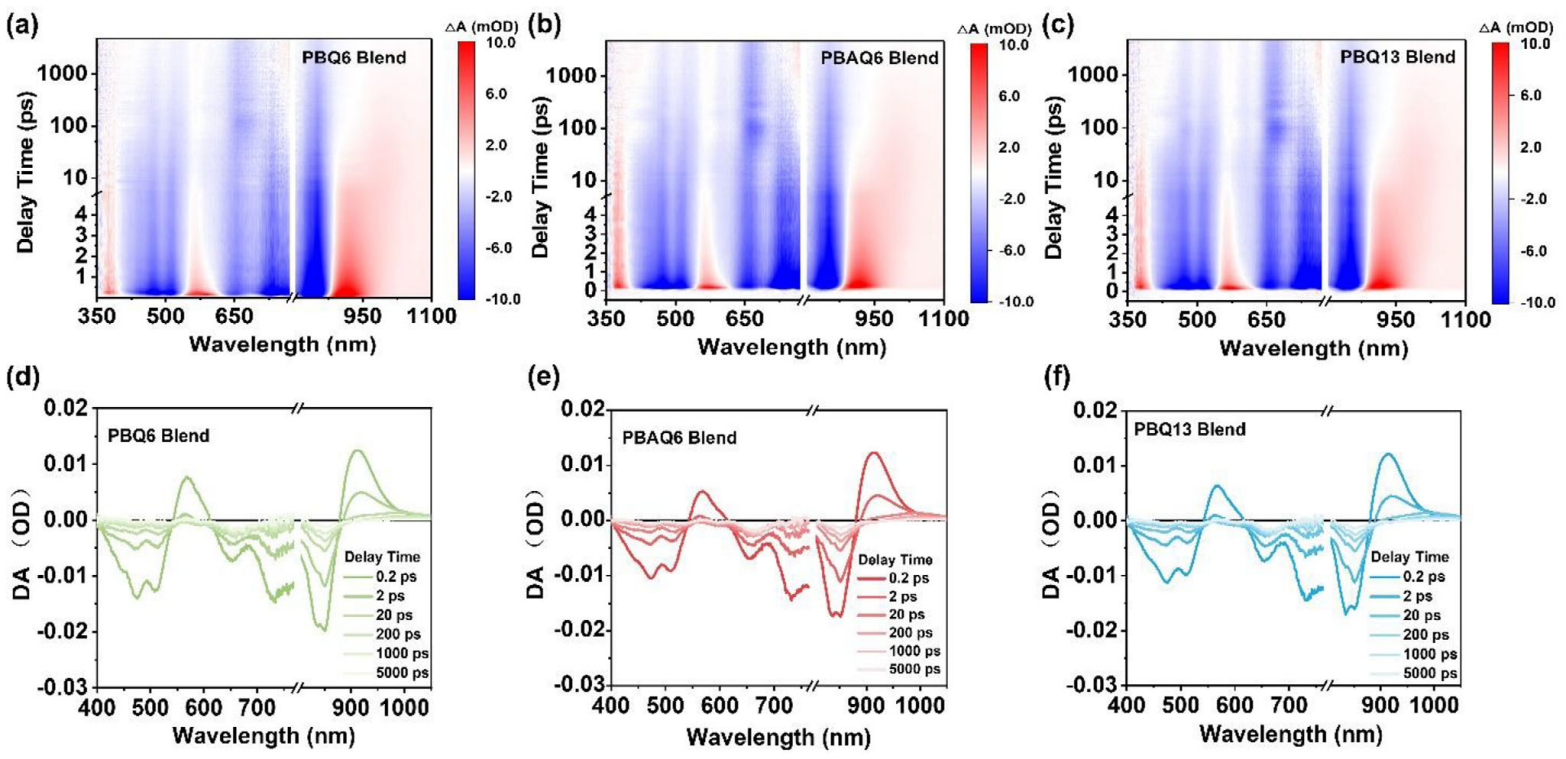
(a–c) Femtosecond transient absorption (fs‐TA) 2D color maps (ΔA) of the PBQ6:PYF‐T‐*o*, PBAQ6:PYF‐T‐*o*, and PBQ13:PYF‐T‐*o* blend films, respectively. (d–f) Corresponding TA spectra extracted at selected delay times for the PBQ6‐, PBAQ6‐, and PBQ13‐based blend films, respectively.

To gain mechanistic insight into how side‐chain asymmetry‐enabled reasonable miscibility regime translates into charge‐generation and decay dynamics, femtosecond transient absorption (TA) spectroscopy was carried out for the PBQ6:PYF‐T‐*o*, PBAQ6:PYF‐T‐*o*, and PBQ13:PYF‐T‐*o* blend films under 810 nm pump to excite the acceptor (Figure 5a–f). The three blends exhibit highly similar early‐time spectral profiles, indicating comparable primary photoexcitation. At an ultrashort delay (∼0.2 ps), the spectra are dominated by excited‐state features of the acceptor excitons, including pronounced ground‐state bleach (GSB) bands (e.g., around ∼500, ∼750, and ∼850 nm) and excited‐state absorption (ESA) bands (e.g., near ∼560 and ∼920 nm). With increasing delay time, these excited‐state ESA signatures decay rapidly, concomitant with the emergence and subsequent enhancement of charge‐related signals, consistent with ultrafast interfacial charge transfer or the formation of charge‐separated (CT or CS) species. Notably, the PBAQ6:PYF‐T‐*o* blend displays a more pronounced donor‐associated bleach band at ∼645–650 nm, suggesting a higher yield of interfacial charge transfer. This interpretation is further supported by global analysis of the kinetic traces (Figure S24 and Table S11), which resolves three characteristic time constants: a sub‐2 ps component (τ_1_) associated with ultrafast interfacial processes, a tens‐of‐ps component (τ_2_) related to diffusion‐assisted charge generation involving the evolution of charge‐transfer‐state populations, and a long‐lived decay component (τ_3_) reflecting the recombination dynamics of charge‐separated species. Importantly, PBAQ6:PYF‐T‐*o* exhibits the longest slow component (τ_3_ = 5620 ± 437 ps), indicative of suppressed charge recombination and an increased probability of charge extraction. Consistently, kinetic comparisons at 645 nm, corresponding to the donor‐associated bleach, and at 997 nm, assigned to charge‐induced ESA associated with photogenerated polaronic species and charge‐separated carriers, further corroborate more persistent charge signatures for the PBAQ6‐based blend (Figure S25). Taken together with the surface‐energy‐derived interaction parameter (relative χ) and the refined‐yet‐continuous morphology revealed by morphology investigations, these TA results support that PBAQ6:PYF‐T‐*o* resides in an appropriate miscibility regime, enabling efficient interfacial charge generation while mitigating non‐productive recombination—thereby contributing to the enhanced device performance, particularly the improved *FF* and *J*_sc_.

## 3 Conclusions

In summary, we developed two quinoxaline‐based D–A copolymer donors (PBAQ6 and PBQ13) and benchmarked them against PBQ6, establishing a clear structure–miscibility–morphology–performance relationship in all‐PSCs. Side‐chain asymmetry in PBAQ6 leads to a more moderate donor–acceptor affinity with PYF‐T‐*o*, as suggested by the contact‐angle‐derived relative χ, and contributes to a favorable balance among blend morphology, molecular packing, charge transport, and recombination. Consequently, the PBAQ6:PYF‐T‐*o* devices achieve more balanced charge transport, enhanced exciton dissociation and charge collection, and reduced recombination losses with a longer carrier lifetime, which together support the enhanced *FF* and *J_sc_*_._ Using non‐halogenated processing, the binary device based on PBAQ6:PYF‐T‐*o* delivers a PCE of 18.35%, and a ternary strategy further boosts the efficiency to 19.52%. These results highlight asymmetric side‐chain design as an effective molecular approach to optimize miscibility and morphology, and identify PBAQ6 as a promising donor for high‐performance all‐PSCs.

## Author Contributions

G. L., Y. L., and C. Z. conceived the project and developed the research idea. C. Z. and B. Q. carried out the synthesis. C. Z. and H. K. performed the photovoltaic measurements and collected the device performance data. J. Y. Z. carried out the transient absorption (TA) measurements and analyzed the data. J. Q. Z. performed the GIWAXS measurements. S. Q. conducted the DFT calculations. H. Z. and Y. C. carried out the contact‐angle measurements. W. L. performed the gel permeation chromatography (GPC) measurements. C. Z., S. L., B. Q. and J. G. wrote the manuscript. S. L. and L. M. revised the manuscript and provided financial support.

## Conflicts of Interest

The authors declare no conflicts of interest.

## Supporting information




**Supporting File 1**: advs77555‐sup‐0001‐SuppMat.docx.

**Supporting File 2**: advs77555‐sup‐0002‐SuppMat.docx.

## Data Availability

The data that support the findings of this study are available from the corresponding author upon reasonable request.
