# Supplementary material for: Side‐Chain Asymmetry in Quinoxaline Copolymer Donors Enables Optimal Morphology and Improved Charge Dynamics for High‐Efficiency Organic Solar Cells
Source: Adv Sci (Weinh). 2026 Sep 6:e77555. Online ahead of print. doi: 10.1002/advs.77555 (PMC13547544; doi:10.1002/advs.77555)
Supplement: Supplementary file 1 [file ADVS-9999-e77555-s001.docx]

**Side-Chain Asymmetry in Quinoxaline Copolymer Donors Enables Optimal Morphology and Improved Charge Dynamics for High-Efficiency Organic Solar Cells**

Can Zhu^a,b,c^, Jinyuan Zhang^c^, Shirong Lu ^a*^, Jianqi Zhang^d^, Shucheng Qin^c^, Ke Hu^c^, Hong Zhang^a^, Yule Cheng^a^, Wenbin Lai^a,c^, Beibei Qiu^e*^, Jing Guo^f*^, Lei Meng^c^, Gang Li ^b *^, Yongfang Li^c *^

^a^ Key Laboratory for Island Green Energy and New Materials, School of Materials Science and Engineering, Taizhou University, Taizhou 318000, China.

^b^ Department of Electrical and Electronic Engineering, Research Institute for Smart Energy (RISE), Photonic Research Institute (PRI), The Hong Kong Polytechnic University, Kowloon, Hong Kong, China.

^c^ Beijing National Laboratory for Molecular Sciences, CAS Key Laboratory of Organic Solids, Institute of Chemistry, Chinese Academy of Sciences, Beijing 100190, China

^d^ CAS Key Laboratory of Nanosystem and Hierarchical Fabrication, National Center for Nanoscience and Technology, Beijing 100190, P. R. China

^e^ Key Laboratory of Solid State Optoelectronic Devices of Zhejiang Province, College of Physics and Electronic Information Engineering, Zhejiang Normal University, Jinhua, Zhejiang 321004, China.

^f^ Province-Ministry Co-construction Collaborative Innovation Center of Hebei Photovoltaic Technology, Hebei Key Laboratory of Optic-electronic Information and Materials, College of Physics Science and Technology, Hebei University, Baoding, Hebei 071002, China.

*Correspondence: lushirong@tzc.edu.cn (S. Lu), qiubeibei@zjnu.edu.cn (B. Qiu), guojing@hbu.edu.cn (J. Guo), gang.w.li@polyu.edu.hk (G. Li), liyf@iccas.ac.cn (Y. Li)

**Experimental Section**

**1. Synthetic Procedures of PBAQ6 and PBQ13**

**Synthesis of the Polymer Acceptor (A) Unit^[1-3]^**

| **Compound** | **Recommended chemical name** |
| --- | --- |
| 1 | 3-Bromothiophene |
| 2 | 3-Bromo-2-alkylthiophene |
| 3 | 3-Bromo-2-alkyl-5-(trimethylsilyl)thiophene |
| 4 | 3-Fluoro-2-alkyl-5-(trimethylsilyl)thiophene |
| 5 | 3-Fluoro-2-alkylthiophene |
| 6 | 5-Bromo-3-fluoro-2-alkylthiophene |
| 7 | 3-Fluoro-2-alkyl-5-[(trimethylsilyl)ethynyl]thiophene |
| 8 | 5-Ethynyl-3-fluoro-2-alkylthiophene |
| 9 | 1-[4-Fluoro-5-(2-ethylhexyl)thiophen-2-yl]-2-[4-fluoro-5-(2-butyloctyl)thiophen-2-yl]ethyne |
| 10 | 1-[4-Fluoro-5-(2-ethylhexyl)thiophen-2-yl]-2-[4-fluoro-5-(2-butyloctyl)thiophen-2-yl]ethane-1,2-dione |
| 11 | 4,7-Dibromo-5,6-difluoro-2,1,3-benzothiadiazole |
| 12 | 3,6-Dibromo-4,5-difluorobenzene-1,2-diamine |
| 13 | 5,8-Dibromo-6,7-difluoro-2-[4-fluoro-5-(2-ethylhexyl)thiophen-2-yl]-3-[4-fluoro-5-(2-butyloctyl)thiophen-2-yl]quinoxaline |
| 14 | 6,7-Difluoro-2-[4-fluoro-5-(2-ethylhexyl)thiophen-2-yl]-3-[4-fluoro-5-(2-butyloctyl)thiophen-2-yl]-5,8-bis(thiophen-2-yl)quinoxaline |
| 15 | 5,8-Bis(5-bromothiophen-2-yl)-6,7-difluoro-2-[4-fluoro-5-(2-ethylhexyl)thiophen-2-yl]-3-[4-fluoro-5-(2-butyloctyl)thiophen-2-yl]quinoxaline |

For compounds 2-8, the alkyl substituent (R) represents either 2-ethylhexyl (R_1_, EH) or 2-butyloctyl (R₂, BO), as indicated in Scheme S1.

**Scheme S1.** Synthetic route to monomer 15 and the corresponding intermediates. The identities of compounds 1–15 is summarized below.

**Synthetic Procedures**

Unless otherwise stated, all reactions involving air- or moisture-sensitive reagents were carried out in oven-dried glassware under an argon atmosphere. Anhydrous THF and toluene were used for organolithium and palladium-catalyzed coupling reactions. Thin-layer chromatography was used to monitor the reaction progress when applicable. The crude products were purified by column chromatography on silica gel or by Soxhlet extraction as described below.

Compounds 1–8 were synthesized according to previously reported procedures for the corresponding alkyl-substituted and fluorinated thiophene building blocks and their ethynyl derivatives.^[4-7]^

**Synthesis of compound 9**

Compound 8-Rₐ (8-EH; 1.86 g, 7.80 mmol, 1.00 equiv), compound 6-Rᵦ (6-BO; 2.90 g, 8.20 mmol, 1.05 equiv), Pd(PPh₃)₄ (0.28 g, 0.25 mmol, 0.03 equiv), and CuI (0.16 g, 0.82 mmol, 0.10 equiv) were added to a dry Schlenk flask. The flask was evacuated and backfilled with argon three times. Degassed THF (30 mL) and triethylamine (10 mL) were then added, and the reaction mixture was stirred at 70 °C for 24 h under argon. After completion of the reaction, the mixture was cooled to room temperature, diluted with dichloromethane, and filtered through a pad of Celite. The filtrate was washed successively with water and brine, dried over anhydrous Na₂SO₄, filtered, and concentrated under reduced pressure. The crude product was purified by silica gel column chromatography using n-hexane to afford compound 9 as a brownish-yellow oil (3.36 g, 85%).

For the asymmetric precursor, Rₐ and Rᵦ represent different alkyl substituents, whereas identical alkyl substituents were used for the corresponding symmetric analogue. The coupling follows a literature-established Sonogashira-type transformation.^[7]^

**Synthesis of compound 10**

Compound 9 (9-asym; 3.36 g, 6.60 mmol, 1.00 equiv) was dissolved in DMSO (25 mL), followed by the addition of iodine (I₂; 3.36 g, 13.2 mmol, 2.00 equiv). The reaction mixture was heated at 150 °C for 12 h under an argon atmosphere. After the starting material was completely consumed, the reaction mixture was cooled to room temperature and poured into water. An aqueous Na₂S₂O₃ solution was added to remove residual iodine, and the mixture was extracted with dichloromethane. The combined organic layers were washed successively with water and brine, dried over anhydrous Na₂SO₄, filtered, and concentrated under reduced pressure. The crude product was purified by silica gel column chromatography using n-hexane/dichloromethane (5:1, v/v)) to afford compound 10 as a light bright-yellow oil (1.07 g, 2.00 mmol, ca. 30%). The oxidation of internal alkynes to the corresponding 1,2-diketones using an I₂/DMSO system has been previously reported.^[8]^

**Compound 11**

Compound 11 was purchased from Energie Chemicals, Inc.

**Synthesis of compound 12**

Compound 11 (0.99 g, 3.00 mmol, 1.00 equiv) was suspended in ethanol (30 mL) under an argon atmosphere and cooled to 0 °C. NaBH₄ (1.13 g, 30.0 mmol, 10.0 equiv) was added portionwise. The reaction mixture was allowed to warm to room temperature and stirred for 5 h until compound 11 was completely consumed. The reaction was carefully quenched with water and extracted with dichloromethane. The combined organic layers were washed successively with water and brine, dried over anhydrous Na₂SO₄, filtered, and concentrated under reduced pressure. Compound 12 was obtained as an off-white solid (0.77 g, ca. 85%) and used directly in the subsequent reaction without further purification.^[9]^

**Synthesis of compound 13**

Compound 10 (10-asym; 1.07 g, 2.00 mmol, 1.00 equiv) and compound 12 (0.77 g, ~2.60 mmol, 1.30 equiv) were dissolved in acetic acid (30 mL) under an argon atmosphere. The reaction mixture was heated at 120 °C for 24 h. After completion of the condensation reaction, the mixture was cooled to room temperature and poured into water. The resulting mixture was extracted with dichloromethane, and the combined organic layers were washed successively with saturated aqueous NaHCO₃, water, and brine. The organic layer was dried over anhydrous Na₂SO₄, filtered, and concentrated under reduced pressure. The crude product was purified by silica gel column chromatography using n-hexane/dichloromethane (8:1, v/v) to afford compound 13 as a pale-yellow solid (1.44 g, 90%). The formation of the quinoxaline core by condensation of a 1,2-diketone with an *o*-phenylenediamine follows well-established procedures for fluorinated quinoxaline monomers.^[10]^

**Synthesis of compound 14**

Compound 13 (13-asym; 1.44 g, 1.80 mmol, 1.00 equiv), 2-(trimethylstannyl)thiophene (1.61 g, 4.32 mmol, 2.40 equiv), and Pd(PPh₃)₄ (0.11 g, 0.09 mmol, 0.05 equiv) were added to a dry pressure tube. The tube was evacuated and backfilled with argon three times. Degassed toluene (25 mL) was added, and the reaction mixture was heated at 110 °C for 24 h under argon. After completion of the reaction, the mixture was cooled to room temperature and the resulting precipitate was collected by filtration and subsequently purified by silica gel column chromatography using n-hexane/dichloromethane (10:1, v/v) to afford compound 14 as a reddish-yellow solid (1.29 g, 89%). This transformation follows the well-established Pd-catalyzed Stille cross-coupling of aryl bromides with organostannyl thiophene derivatives.^[11, 12]^

**Synthesis of monomer 15**

Compound 14 (14-asym; 1.29 g, 1.60 mmol, 1.00 equiv) bearing asymmetric alkyl substituents was dissolved in a mixture of chloroform (20 mL) and DMF (3 mL) under an argon atmosphere, and the solution was cooled to 0 °C. NBS (0.63 g, 3.50 mmol, 2.20 equiv) was added portionwise. The reaction mixture was stirred at 0 °C for 1 h and then allowed to warm to room temperature. After completion of the reaction, the mixture was diluted with dichloromethane and washed successively with water and brine. The organic layer was dried over anhydrous Na₂SO₄, filtered, and concentrated under reduced pressure. The crude product was purified by silica gel column chromatography using n-hexane to afford monomer 15 as an orange-red solid (1.39 g, 90%).

**¹H NMR** (400 MHz, CDCl₃, 298 K): δ 7.68–7.66 (m, 2H), 7.20 (d, *J* = 4.0 Hz, 2H), 7.12–7.11 (m, 2H), 2.84–2.80 (m, 4H), 1.80–1.69 (m, 2H), 1.52–1.20 (m, 24H), 1.03–0.88 (m, 12H) ppm.

**¹³C {^1^H }NMR** (100 MHz, CDCl₃, 298 K): δ 154.12 (d, ¹*J*(C,F) ≈ 254 Hz), 149.58 (d, ¹*J*(C,F) ≈ 258 Hz), 149.38 (d, ¹*J*(C,F) ≈ 255 Hz), 143.70, 134.24, 133.35, 131.05, 130.97, 129.43, 127.55, 119.95, 118.81, 116.77, 40.84, 39.36, 35.69, 33.63, 32.51, 31.94, 29.83, 29.74, 29.67, 29.24, 28.91, 26.83, 25.76, 25.09, 22.73, 14.31, 14.24, 14.13, 11.01, 10.92 ppm.

**¹⁹F NMR** (564.63 MHz spectrometer, CDCl₃, 298 K): δ −128.19, −130.78, −130.82 ppm.

**HRMS (MALDI-FT-ICR)** *m/z*: [M]•⁺ calcd for C₄₄H₄₈Br₂F₄N₂S₄, 966.0998; found, 966.0991.

**Synthesis of monomer 17**

The corresponding symmetrically alkyl-substituted precursor **14-sym** (1.13 g, 1.30 mmol, 1.00 equiv) was dissolved in a mixture of chloroform (22 mL) and DMF (4 mL) under an argon atmosphere, and the solution was cooled to 0 °C. N-Bromosuccinimide (NBS; 0.51 g, 2.86 mmol, 2.20 equiv) was added portionwise. The reaction mixture was stirred at 0 °C for 1 h and then allowed to warm to room temperature and stirred until completion of the reaction. The mixture was diluted with dichloromethane and washed successively with water and brine. The organic layer was dried over anhydrous Na₂SO₄, filtered, and concentrated under reduced pressure. The crude product was purified by silica gel column chromatography using *n-hexane* as the eluent to afford monomer 17 as an orange-red solid (1.24 g, 93%).

**¹H NMR** (400 MHz, CDCl₃, 298 K): δ 7.47 (d, *J* = 4.0 Hz, 2H), 7.06 (s, 2H), 6.95 (d, *J* = 4.0 Hz, 2H), 2.70–2.68 (m, 4H), 1.68–1.65 (m, 2H), 1.31–1.21 (m, 32H), 0.85–0.78 (m, 12H) ppm.

**¹³C {^1^H} NMR** (100 MHz, CDCl₃, 298 K): δ 154.07 (d, ¹*J*(C,F) ≈ 254 Hz), 149.34 (m, C–F coupled), 143.51, 143.49, 134.17, 134.09, 133.30, 133.15, 133.11, 131.86, 131.12, 131.01, 130.93, 130.85, 130.74, 130.24, 127.48, 127.30, 118.83, 118.56, 118.47, 118.35, 118.23, 116.60, 116.54, 116.50, 39.38, 33.48, 33.01, 31.98, 29.73, 29.71, 28.84, 26.65, 23.04, 22.76, 14.23, 14.20 ppm.

**¹⁹F NMR** (564.63 MHz spectrometer, CDCl₃, 298 K): δ −128.28, −130.79 ppm.

**HRMS (MALDI-FT-ICR)** m/z: [M]•⁺ calcd for C₄₈H₅₆Br₂F₄N₂S₄, 1022.1624; found, 1022.1618.

**Synthesis of PBAQ6**

Monomer 15 (67.80 mg, 0.070 mmol, 1.00 equiv), monomer 16 (65.84 mg, 0.070 mmol, 1.00 equiv), and Pd(PPh₃)₄ (1.8 mg, 0.00156 mmol, 2.2 mol%) were charged into a pressure tube. Toluene (5.0 mL) and DMF (0.10 mL) were added, and the reaction mixture was stirred at 115 °C for 23 h under an argon atmosphere. After cooling to room temperature, the reaction mixture was slowly poured into methanol (100 mL) under vigorous stirring. The resulting precipitate was collected by filtration and washed thoroughly with methanol. The crude polymer was purified by Soxhlet extraction successively with methanol, n-hexane, and chloroform. The chloroform fraction was concentrated and reprecipitated into methanol. The resulting polymer was collected by filtration and dried under vacuum to afford PBAQ6 as a dark-colored solid (37 mg). ¹H NMR (400 MHz, o-dichlorobenzene-*d*₄, 393 K): δ 8.11–7.28 (br, aromatic H), 2.92–2.82 (br, alkyl CH₂ adjacent to the thiophene units), 1.83–0.78 (br, aliphatic H) ppm. ¹⁹F NMR (659 MHz, CDCl₃): δ −128.00, −130.5 to −132.8 (br) ppm.

**Scheme S2.** Synthetic route of the polymer PBAQ6.

**Synthesis of PBQ13**

Monomer 17 (71.75 mg, 0.071 mmol, 1.00 equiv), monomer 16 (67.07 mg, 0.071 mmol, 1.00 equiv), and Pd(PPh₃)₄ (1.7 mg, 0.00147 mmol, 2.1 mol%) were added to a dry pressure tube under an argon atmosphere. Anhydrous toluene (5.0 mL) was then added, and the reaction mixture was stirred at 115 °C for 36 h. After cooling to room temperature, the reaction mixture was slowly poured into methanol (100 mL) under vigorous stirring. The resulting precipitate was collected by filtration and washed thoroughly with methanol.

The crude polymer was purified by Soxhlet extraction successively with methanol, n-hexane, and chloroform. The chloroform fraction was concentrated and reprecipitated into methanol. The resulting polymer was collected by filtration and dried under vacuum to afford PBQ13 as a dark-colored solid (43 mg). ¹H NMR (400 MHz, o-dichlorobenzene-*d*₄, 393 K): δ 8.11–7.23 (br, aromatic H), 2.92–2.85 (br, alkyl CH₂ adjacent to the thiophene units), 1.83–0.79 (br, aliphatic H) ppm. ¹⁹F NMR (659 MHz, CDCl₃): δ −127.95, −130.5 to −132.3 (br) ppm.

**Scheme S3.** Synthetic route of the polymer PBQ13.

**2. Characterization of materials**

¹H and ¹³C NMR spectra were recorded on a Bruker AV-400 spectrometer in CDCl₃ at 298 K, unless otherwise specified. ¹⁹F NMR spectra of monomers 15 and 17 were recorded at 564.63 MHz in CDCl₃ at 298 K, whereas those of PBAQ6 and PBQ13 were recorded at 658.76 MHz in CDCl₃ at 298 K. For ¹H and ¹³C NMR spectra, chemical shifts were referenced to tetramethylsilane (TMS). High-resolution mass spectra (HRMS) were acquired using a Bruker solariX MALDI-FT-ICR mass spectrometer in positive-ion mode. Molecular weights were determined by high-temperature gel permeation chromatography (GPC) on an Agilent PL-GPC 220 system using o-dichlorobenzene as the eluent at 140 °C. The GPC system was equipped with PLgel Olexis columns and calibrated with polystyrene standards. Polymer samples were dissolved in o-dichlorobenzene at a concentration of 1.0 mg mL^−1^ and filtered through a 0.45 μm PTFE filter before injection. UV–vis absorption spectra were collected on a Hitachi U-3010 spectrophotometer. For solution measurements, PBQ6, PBAQ6, PBQ13, and PYF-T-*o* were dissolved in toluene at a concentration of 0.1 mg mL^−1^, and the corresponding spectra were normalized for comparison. For thin-film measurements, neat donor polymer films were prepared by spin-coating toluene solutions of PBQ6, PBAQ6, and PBQ13 with a concentration of 8 mg mL^−1^ onto quartz substrates at 2200 rpm for 30 s. The corresponding blend films were prepared from toluene solutions with a total concentration of 16 mg mL^−1^ and spin-coated onto quartz substrates under the same conditions. The films were treated under the same thermal annealing conditions as those used for device fabrication, unless otherwise stated. The absorption coefficients of the thin films were calculated from the measured absorbance spectra and film thicknesses.

Cyclic voltammetry (CV) was carried out using a Zahner IM6e electrochemical workstation with a glassy carbon working electrode, a platinum wire counter electrode, and an Ag/AgCl reference electrode. Measurements were performed in 0.1 M tetrabutylammonium hexafluorophosphate (Bu₄NPF₆) acetonitrile electrolyte at a scan rate of 50 mV s⁻¹. The ferrocene/ferrocenium (Fc/Fc⁺) redox couple was used as an internal standard.

The surface energies of the polymer films were calculated using the Owens–Wendt–Rabel–Kaelble (OWRK) method from the contact angles of water and diiodomethane, in which the total surface energy is separated into dispersive and polar components.^[13]^

**3. Simulation details.**

All calculations are performed by ORCA (version 6.1.0) unless otherwise stated.^[14]^ The geometries were optimized by DFT calculation under B3LYP/def2-SVP level. The molecular orbitals and geometries were analyzed by Multiwfn (version 3.8) and VMD (version 2.0.0a7) for visualization.^[15, 16]^

**4.** **Fabrication and characterization of OSCs**

All OSCs were fabricated in a conventional architecture of ITO/PEDOT:PSS/active layer/PDINN/Ag. Pre-patterned ITO glass substrates (sheet resistance = 10 Ω sq⁻¹) were sequentially sonicated in detergent, deionized water, acetone, and isopropanol, followed by UV–ozone treatment for 25 min (Jelight, USA). PEDOT:PSS (Baytron P 4083, HCStarck) was filtered through a 0.45 μm filter and spin-coated onto the cleaned ITO at 5000 rpm for 30 s, and the films were then annealed in air at 150 °C for 0.5 h. 2PACz was dissolved in ethanol at a concentration of 0.3 mg mL^-1^, then poured onto ITO at 3000 rpm and annealed at 100 °C for 10 min. The polymer donor: PYF-T-*o* blend (D:A = 1:1.1; total concentration 18 mg mL⁻¹) was prepared in toluene (when using 2PACz as the hole transport layer, chloroform is used as the solvent.) with 1-chloronaphthalene (CN, 0.7 vol%), DTT, tBP and DIB as an additive. The active-layer solution was spin-coated onto the PEDOT:PSS layer at 2400 rpm for 30 s, followed by thermal annealing at 95 °C-100 °C for 5 min. Subsequently, a PDINN cathode interlayer was deposited by spin-coating a methanol solution of PDINN (1.0 mg mL⁻¹) at 5000 rpm for 30 s. After cooling to room temperature, the substrates were transferred to a vacuum chamber, and Ag (≈100 nm) was thermally evaporated under 1 × 10⁻⁵ Pa. The active area of the devices was 6.0 mm².

The current density–voltage (*J–V*) characteristics were measured in a nitrogen-filled glovebox (O₂ and H₂O < 0.1 ppm) using a Keithley 2450 source meter under simulated AM 1.5G illumination from an Oriel Sol3A Class AAA solar simulator (Newport 94023A, 450 W xenon lamp). The light intensity was calibrated to 100 mW cm⁻² with a Newport Oriel 91150V reference cell. External quantum efficiency (EQE) spectra were recorded using a QE-R3-011 spectral response system (Enli Technology, Taiwan), with the light intensity at each wavelength calibrated using standard single-crystal silicon photovoltaic cells.

Photo-CELIV and transient photovoltage (TPV) measurements were performed on devices fabricated identically to those described above, using an integrated characterization platform (Paios, Fluxim AG, Switzerland). For photo-CELIV, the delay time was set to 0 s, the light intensity to 100%, and the light-pulse duration to 100 μs; the voltage ramp rate was varied from 20 to 100 V ms⁻¹. For TPV, the background light intensity was set to 0.10%, 0.23%, 0.53%, 1.23%, 2.83%, 6.52%, 15.0%, 34.6%, and 80.0%, with a relative pulse intensity of 20.0%; the settling time was 30.0 ms, the pulse length was 5.0 ms, and the follow-up time was 30.0 μs. In the TPC measurement, the light intensity is 10%, 17.8%, 31.6%, 56.2% and 100%, respectively, rising from 0.1 sun to 1 sun. The settling time is 100 µs, pulse length is 100 µs and the follow-up time is 200 µs.

**5. Hole mobility and electron mobility measurements**

ITO/PEDOT:PSS/active layer/MoO_3_/Ag device structure is used to test hole mobility, and ITO/ZnO/active layer/PDINN/Al is used to test electron mobility. The hole and electron mobilities are calculated according to the space charge limited current (SCLC) method with the equation: *J* = 9µɛ_r_ɛ_0_*V*^2^ /8*d*^3^, where *J* is the current density, µ is the hole or electron mobility, *V* is the internal voltage in the device, ε_r_ is the relative dielectric constant of active layer material, ε_0_ is the vacuum permittivity, and *d* is the thickness of the active layer.^[17]^

**6.** **GIWAXS measurements.**

The samples for the GIWAXS measurements were prepared on Si substrates from the corresponding solutions in toluene. The neat-film samples were prepared using individual-material solutions at a concentration of 8 mg mL⁻¹, whereas the blend-film samples were prepared using the corresponding blend solutions with a total solute concentration of 16 mg mL⁻¹. A 10 keV X-ray beam was incident on the samples at a grazing angle of 0.13–0.17°. The scattered X-rays were recorded using a Dectris Pilatus 2M photon-counting detector.

**7. Atomic force microscopy (AFM) and Transmission electron microscopy (TEM)**

Atomic force microscopy (AFM) was employed to probe the surface morphology of the blend films. The samples were prepared by spin-coating the polymer donor:PYF-T-*o* blend solutions onto silicon substrates under the same processing conditions as those used for device fabrication. AFM measurements were carried out on a Bruker Dimension Icon system in tapping (intermittent-contact) mode using a standard silicon cantilever with a nominal resonance frequency of ~120 kHz. Both height and phase images were collected, and the root-mean-square roughness (Rq) values were extracted from the height images.

Transmission electron microscopy (TEM) was used to examine the nanoscale phase morphology of the blend films. For TEM sample preparation, the active-layer films were spin-coated onto a sacrificial substrate, released by floating in deionized water, and then transferred onto copper grids for imaging. TEM images were acquired on a JEOL JEM-2100 microscope operated at an accelerating voltage of 200 kV in bright-field mode. No staining treatment was applied to the samples. Quantitative grayscale analysis of the TEM images was performed using ImageJ.^[18]^ The original TEM images were converted to 8-bit grayscale images before analysis. For each blend film, an identical 350 × 350 pixel region of interest was selected from the central area of the TEM image, while avoiding labels, scale bars, and image edges. The mean gray value, standard deviation, coefficient of variation, and normalized RMS contrast were extracted to compare the image uniformity, local intensity fluctuations, and contrast among the three blend films.

**8. Transient absorption spectroscopy**

A Ti:sapphire regenerative amplifier (Astrella, Coherent) delivered 800 nm pulses with a duration of ~35 fs at a repetition rate of 1 kHz and a maximum pulse energy of 7 mJ. The amplifier output was divided into two beam paths using a beam splitter. One portion was used to pump an optical parametric amplifier (TOPAS C, Light Conversion) to generate the excitation (pump) pulses, while the remaining portion was sent to an ultrafast transient absorption setup (HARPIA-TA, Light Conversion) to produce a white-light continuum probe. In the spectrometer, the pump beam, modulated at 500 Hz by a chopper, was spatially and temporally overlapped with the probe on the sample. The pump fluence was maintained at 2 μJ cm⁻² to minimize singlet–singlet annihilation. For TA measurements, film samples were prepared by spin-coating the blend solutions onto thin quartz substrates, and the resulting films were pre-annealed at 90 °C for 5 min under nitrogen prior to data acquisition.

**Figure S1.** ^1^H NMR spectrum of monomer **15** **(PBAQ6)** in CDCl_3._

**Figure S2.** ^13^C NMR spectrum of monomer **15 (PBAQ6)** in CDCl_3._

**Figure S3.** ^19^F NMR spectrum of monomer **15 (PBAQ6).**

**Figure S4.** High-resolution MALDI-FT-ICR mass spectrum of monomer 15 **(PBAQ6)**.

**Figure S5.** ¹H NMR spectrum of PBAQ6 recorded at 400 MHz in o-dichlorobenzene-*d*₄ at 393 K.

**Figure S6.** ¹^9^F NMR spectrum of PBAQ6 recorded at 658.76 MHz in CDCl₃ at 298 K.

**Figure S7.** ^1^H NMR spectrum of monomer **17 (PBQ13)** in CDCl_3._

**Figure S8** ^13^C NMR spectrum of monomer **17 (PBQ13)** in CDCl_3._

**Figure S9.** ^19^F NMR spectrum of monomer **17 (PBQ13).**

**Figure S10.** High-resolution MALDI-FT-ICR mass spectrum of monomer 17 **(PBQ13)**.

**Figure S11.** ¹H NMR spectrum of PBQ13 recorded at 400 MHz in o-dichlorobenzene-*d*₄ at 393 K.

**Figure S12.** ¹^9^F NMR spectrum of PBQ13 recorded at 658.76 MHz in CDCl₃ at 298 K.

**Figure S13**. GPC chart relates to the molecular weight of PBQ6 (a), PBAQ6 (b) PBQ13 (c) and PYF-T-*o* (d).

**Figure S14**. (a) Normalized UV–vis absorption spectra of PBQ6, PBAQ6, PBQ13, and PYF-T-o in toluene solution. (b) Absorption-coefficient spectra of neat donor polymer films prepared from 8 mg mL^−1^ toluene solutions. (c) Normalized UV–vis absorption spectra of PBQ6, PBAQ6, and PBQ13 blend films. (d) Absorption-coefficient spectra of the corresponding blend films prepared from toluene solutions with a total concentration of 16 mg mL^−1^.

**Figure S15**. Cyclic voltammograms of the polymers PBQ6 (a), PBAQ6 (b) PBQ13 (c) and PYF-T-*o* (d).

**Figure S16**. Cyclic voltammograms of the Fc/Fc^+^.

**Figure S17.** DFT-calculated frontier molecular orbital distributions of PBQ6, PBAQ6, and PBQ13 based on extended oligomer models containing four repeating units.

**Figure S18.** AFM height images of the PBAQ6:PYF-T-*o* (a) and PBQ13:PYF-T-*o* (b) blend films.

**Figure S19**. TEM images of the PBQ6:PYF-T-o (a), PBAQ6:PYF-T-o (b), and PBQ13:PYF-T-o (c) blend films.

**Figure S20.** 2D GIWAXS patterns of PYF-T-*o* neat films, and line cuts of GIWAXS images of the corresponding neat films.

**Figure S21**. *J-V* curves of PBAQ6 and PBQ13 (similar molecular weights) with PEDOT:PSS as HTL.

**Figure S22**. *J*^1/2^~*V* characteristics of the hole mobility measurements of the neat films (a), the hole mobility measurements of the blend films (b), and the electron mobility measurements of the blend films (c) in the dark.

**Figure S23**. Normalized transient photocurrent responses of PBQ6:PYF-T-*o*, PBAQ6:PYF-T-*o*, and PBQ13:PYF-T-*o* devices: (a) photocurrent rise dynamics after switching on the light pulse; (b) photocurrent decay dynamics after switching off the light pulse.

**Figure S24.** Global fitting of kinetic traces of PBQ6, PBAQ6 and PBQ13 blend films.

**Figure S25.** Transient absorption (TA) kinetics of the PBQ6, PBAQ6, and PBQ13 blend films probed at (a) 645 nm and (b) 997 nm, showing the evolution of ΔA as a function of delay time.

**Table S1.** Contact angles of the donor and acceptor materials and Flory−Huggins interaction parameters between the polymer donor and PYF-T-*o* acceptor.

| ***θ* _Water_** | ***θ* _Diiodomethane_** | **γ (mN m^-1^)** | **relative χ ^a^** |
| --- | --- | --- | --- |
|  |  | 40.2 | − |
|  |  | 29.3 | 0.86*K* |
|  |  | 33.0 | 0.35*K* |
|  |  | 42.8 | 0.04*K* |
|  |  | 33.0**^b^** | 0.35*K* **^b^** |
|  |  | 38.0 **^b^** | 0.03*K* **^b^** |

a. The Flory−Huggins interaction parameter (χ) are calculated by$\chi={\text{K}\left( \sqrt{\gamma_{d}}-\sqrt{\gamma_{a}} \right)}^{2}$.

b. Surface energy and χ of the PBAQ6 and PBQ13 approximately similar molecular weights.

**Table S2.** AFM height images and statistical analysis of the root-mean-square roughness (*R*_q_) of the PBQ6:PYF-T-*o*, PBAQ6:PYF-T-*o*, and PBQ13:PYF-T-*o* blend films.

| **Sample** | | | **Mean ± Standard deviation (nm)** |
| --- | --- | --- | --- |
|  |  |  | **1.00 ± 0.04** |
|  |  |  | **0.87 ± 0.02** |
|  |  |  | **0.78 ± 0.04** |

**Table S3.** Quantitative grayscale analysis of the TEM images of the corresponding blend films.

| **Blend film** | **Area/pixels** | **Mean gray value** | **StDev** | **CV** | **Normalized RMS contrast/%** |
| --- | --- | --- | --- | --- | --- |
| **PBQ6:PYF-T-*o*** | 122500 | 136.8 | 29.3 | 0.214 | 21.43 |
| **PBAQ6:PYF-T-*o*** | 122500 | 140.1 | 27.0 | 0.193 | 19.30 |
| **PBQ13:PYF-T-*o*** | 122500 | 136.3 | 26.0 | 0.191 | 19.09 |

**Table S4.** GIWAXS measurement parameters of the related films.

| **Samples** | **q 100 peak [1/Å]** | **Lamellar spacing [Å]** | **Lamellar peak FWHM [1/Å]** | **Coherence length [Å]** | **q 010 peak [1/Å]** | **π-π spacing [Å]** | **π-π peak FWHM [1/Å]** | **Coherence length [Å]** |
| --- | --- | --- | --- | --- | --- | --- | --- | --- |
| **PBQ6** | 0.254 | 24.712 | 0.098 | 57.96 | 1.704 | 3.69 | 0.228 | 25.11 |
| **PBAQ6** | 0.266 | 23.659 | 0.085 | 66.58 | 1.684 | 3.731 | 0.253 | 22.58 |
| **PBQ13** | 0.281 | 22.360 | 0.066 | 86.005 | 1.686 | 3.727 | 0.293 | 19.516 |
| **PYF-T-*o*** | 0.413 | 15.204 | 0.060 | 94.55 | 1.599 | 3.929 | 0.325 | 17.549 |
| **PBQ6:PYF-T-*o*** | 0.259 | 24.290 | 0.079 | 71.80 | 1.672 | 3.759 | 0.266 | 21.514 |
| **PBAQ6:PYF-T-*o*** | 0.264 | 23.811 | 0.098 | 58.01 | 1.631 | 3.853 | 0.318 | 17.965 |
| **PBQ13:PYF-T-*o*** | 0.268 | 23.434 | 0.060 | 94.494 | 1.675 | 3.751 | 0.319 | 17.922 |

**Table S5.** Photovoltaic performance of the all-PSCs based on PBAQ6:PYF-T-*o* with different device fabrication conditions with PEDOT:PSS as HTL, under the illumination of AM 1.5 G, 100 mW cm^-2^.

| **D/A**  **Ratio** | **Concentration (CF)** | **Additive (CN)** (%) | **Annealing**  ( ℃ ) | ***V*_oc_** (V) | ***J*_sc_**  (mA cm^-2^) | **FF** (%) | **PCE** (%) |
| --- | --- | --- | --- | --- | --- | --- | --- |
| 1:0.8 | 16 mg mL^-1^ | 1 | 95 | 0.92 | 25.05 | 74.06 | 17.07 |
|  |  |  | 105 | 0.91 | 25.18 | 73.68 | 16.88 |
| 1:1.1 |  | 0.7 | 85 | 0.92 | 24.41 | 68.89 | 15.47 |
|  |  |  | 95 | 0.91 | 24.53 | 69.32 | 15.47 |
|  |  | 1 | / | 0.92 | 22.54 | 64.18 | 13.31 |
|  |  |  | 85 | 0.91 | 24.76 | 76.22 | 17.17 |
|  |  |  | 95 | 0.91 | 25.02 | 77.01 | 17.53 |
|  |  | 1.5 | 85 | 0.91 | 23.83 | 75.88 | 16.45 |
|  |  |  | 95 | 0.89 | 24.01 | 76.56 | 16.36 |
| 1:1.3 |  | 1 | 95 | 0.90 | 24.56 | 76.81 | 16.97 |
|  |  |  | 105 | 0.90 | 24.81 | 75.63 | 16.89 |

**Table S6.** Photovoltaic performance of the all-PSCs based on PBQ6:PYF-T-*o* with different device fabrication conditions with PEDOT:PSS as HTL, under the illumination of AM 1.5 G, 100 mW cm^-2^.

| **D/A Ratio** | **Concentration (CF)** | **Additive (CN)** (%) | **Annealing**  ( ℃ ) | ***V*_oc_** (V) | ***J*_sc_**  (mA cm^-2^) | **FF** (%) | **PCE** (%) |
| --- | --- | --- | --- | --- | --- | --- | --- |
| 1:1.1 | 16 mg mL^-1^ | **/** | **/** | 0.930 | 22.34 | 66.57 | 13.83 |
|  |  | **/** | 95 | 0.922 | 23.36 | 68.06 | 14.66 |
|  |  | 1 |  | 0.890 | 24.72 | 73.21 | 16.11 |
| 1:1.3 |  |  |  | 0.890 | 24.53 | 73.31 | 16.00 |
|  |  | 1.2 |  | 0.880 | 24.24 | 73.16 | 15.61 |

**Table S7.** Photovoltaic performance parameters of the all-PSCs (with PEDOT:PSS as HTL) based on PBAQ6:PYF-T-*o* (1:1.1, w/w, thermal annealing at 95 ℃ for 5 min) with different additives under the illumination of AM 1.5G, 100 mW cm^-2^.

| **Active layer** | **Additive** | ***V*_oc_**  **(V)** | ***J*_sc_**  **(mA cm^-2^)** | **FF**  **(%)** | **PCE**  **(%)** |
| --- | --- | --- | --- | --- | --- |
| PBAQ6:PYF-T-*o* | CN | 0.910 | 25.02 | 77.01 | 17.53 |
|  | tBP | 0.921 | 24.31 | 68.69 | 15.36 |
|  | DTT | 0.900 | 24.11 | 76.27 | 16.55 |

**Figure S26.** High-temperature GPC curves of the additional PBAQ6-A and PBQ13-A batches.

**Table S8.** High-temperature GPC data for the additional PBAQ6-A and PBQ13-A batches.

| Other batches | Mw (g/mol) | Mn (g/mol) | Mw / Mn | Mz (g/mol) | Mz1 (g/mol) | Mz / Mw | Mv (g/mol) | Mp (g/mol) |
| --- | --- | --- | --- | --- | --- | --- | --- | --- |
| PBAQ6-A | 23300 | 8600 | 2.70 | 42300 | 65700 | 1.82 | 21200 | 21400 |
| PBQ13-A | 25700 | 10000 | 2.56 | 44100 | 64400 | 1.72 | 23500 | 23000 |

**Table S9.** Photovoltaic performance of the all-PSCs based on other batches of PBAQ6 and PBQ13 with PEDOT:PSS as HTL, under the illumination of AM 1.5 G, 100 mW cm^-2^.

| **Blend films** | ***V*_oc_** (V) | ***J*_sc_** (mA cm^-2^) | | **FF** (%) | **PCE** (%) |
| --- | --- | --- | --- | --- | --- |
| PBAQ6:PYF-T-*o* | 0.914 | | 24.93 | 75.93 | 17.30 |
| PBQ13:PYF-T-*o* | 0.918 | | 24.64 | 65.75 | 14.87 |

**Table S10.** Detailed data of the hole mobility (*μ*_h_) and electron mobility (*μ*_e_).

| **Active layer** | ***μ*_e_**（$10$^-4^ cm^2^ V^-1^ s^-1^） | ***μ*_h_**（$10$^-4^ cm^2^ V^-1^ s^-1^） | ***μ*_e_/*μ*_h_** |
| --- | --- | --- | --- |
| PBQ6:PYF-T-*o* | 7.50 | 6.36 | 1.18 |
| PBAQ6: PYF-T-*o* | 7.63 | 6.80 | 1.12 |
| PBQ13:PYF-T-*o* | 2.27 | 1.68 | 1.35 |

**Table S11.** Global fitting parameters ($\tau_{1}$-$\tau_{3}$) of TA kinetic traces for PBQ6:PYF-T-*o*, PBAQ6:PYF-T-*o*, and PBQ13:PYF-T-*o* blend films.

| **Sample** | $\boldsymbol{\tau}_{\boldsymbol{1}}$***^a^* (ps)** | $\boldsymbol{\tau}_{\boldsymbol{2}}$***^b^* (ps)** | $\boldsymbol{\tau}_{\boldsymbol{3}}$***^c^* (ps)** |
| --- | --- | --- | --- |
| **PBQ6 Blend** | 1.22 ±0.06 | 49.1 ± 3.72 | 4601 ± 278 |
| **PBAQ6 Blend** | 1.27 ±0.04 | 52.5 ± 3.91 | 5620 ± 437 |
| **PBQ13 Blend** | 1.24 ±0.04 | 57.3 ± 5.09 | 5060 ± 390 |
| a: Ultrafast interfacial charge transfer.  b: Exciton-diffusion-assisted charge transfer.  c: Charge recombination (decay of long-lived charge-separated species). | | | |

**Reference**

[1]	D.-X. Li, S.-F. Li, C.-L. Wen, C.-Y. Sun, C.-Z. Shi, X.-X. Xia, X.-H. Lu, L.-H. Jiang, J. Yuan, Y.-P. Zou, Quinoxaline-based Polymers with Asymmetric Aromatic Side Chain Enables 16.27% Efficiency for Organic Solar Cells, *Chinese Journal of Polymer Science* 41 (2023): 1002, <https://doi.org/10.1007/s10118-023-2895-5>.

[2]	Z. Zhou, Z. Wu, Y. Xu, Y. Lu, J. Yang, W. Zhu, Y. Liu, Asymmetric fluorine and chlorine side-chain engineered quinoxaline-based D–A copolymer for both fullerene and nonfullerene polymer solar cells, *Polymer Chemistry* 14 (2023): 728, <https://doi.org/10.1039/d2py01122k>.

[3]	D. Li, S. Liang, X. Hua, B. Fan, J. Song, X. Lu, W. Huang, J. Yuan, Y. Zou, Selecting Solid Additives to Control the Interaction between Polymer Donors and Additives for Constructing High-Efficiency Organic Solar Cells, *Macromolecules* 57 (2024): 3580, <https://doi.org/10.1021/acs.macromol.4c00362>.

[4]	Z. Fei, P. Boufflet, S. Wood, J. Wade, J. Moriarty, E. Gann, E. L. Ratcliff, C. R. McNeill, H. Sirringhaus, J. S. Kim, M. Heeney, Influence of Backbone Fluorination in Regioregular Poly(3-alkyl-4-fluoro)thiophenes, *J Am Chem Soc* 137 (2015): 6866, <https://doi.org/10.1021/jacs.5b02785>.

[5]	Q. Zhang, L. Yan, X. Jiao, Z. Peng, S. Liu, J. J. Rech, E. Klump, H. Ade, F. So, W. You, Fluorinated Thiophene Units Improve Photovoltaic Device Performance of Donor–Acceptor Copolymers, *Chemistry of Materials* 29 (2017): 5990, <https://doi.org/10.1021/acs.chemmater.7b01683>.

[6]	J. Huang, R. Peng, L. Xie, W. Song, L. Hong, S. Chen, Q. Wei, Z. Ge, A novel polymer donor based on dithieno[2,3-d:2′,3′-d′′]benzo[1,2-b:4,5-b′]dithiophene for highly efficient polymer solar cells, *Journal of Materials Chemistry A* 7 (2019): 2646, <https://doi.org/10.1039/c8ta11004b>.

[7]	K. Sonogashira, Yasuo Tohda, Nobue Hagihara, A convenient synthesis of acetylenes: catalytic substitutions of acetylenic hydrogen with bromoalkenes, iodoarenes and bromopyridines, *Tetrahedron Letters* 16 (1975): 4467, <https://doi.org/10.1016/S0040-4039(00)91094-3>.

[8]	S. Naidoo, V. Jeena, One‐Pot, Two‐Step Metal and Acid‐Free Synthesis of Trisubstituted Imidazole Derivatives via Oxidation of Internal Alkynes Using an Iodine/DMSO System, *European Journal of Organic Chemistry* 2019 (2019): 1107, <https://doi.org/10.1002/ejoc.201801584>.

[9]	X. Song, M. Wang, L. Kong, J. Zhao, Effects of the acceptor pattern and substitution position on the properties of N-phenyl-carbazolyl based donor–acceptor–donor molecules, *RSC Advances* 7 (2017): 18189, <https://doi.org/10.1039/c7ra01449j>.

[10]	C. Zhu, L. Meng, J. Zhang, S. Qin, W. Lai, B. Qiu, J. Yuan, Y. Wan, W. Huang, Y. Li, A Quinoxaline-Based D-A Copolymer Donor Achieving 17.62% Efficiency of Organic Solar Cells, *Advanced Materials* 33 (2021): e2100474, <https://doi.org/10.1002/adma.202100474>.

[11]	C. Zhu, K. Hu, L. Meng, X. Kong, W. Lai, S. Qin, B. Qiu, J. Zhang, Z. Zhang, Y. Wu, X. Li, Y. Li, High-Performance D–A Copolymer Donor Based on Difluoroquinoxaline A-Unit with Alkyl-Chlorothiophene Substituents for Polymer Solar Cells, *CCS Chemistry* 5 (2023): 2378, <https://doi.org/10.31635/ccschem.022.202202491>.

[12]	K. Hu, C. Zhu, S. Qin, W. Lai, J. Du, L. Meng, Z. Zhang, Y. Li, n-Octyl substituted quinoxaline-based polymer donor enabling all-polymer solar cell with efficiency over 17%, *Science Bulletin (Beijing)* 67 (2022): 2096, <https://doi.org/10.1016/j.scib.2022.10.005>.

[13]	D. K. Owens, R. C. Wendt, Estimation of the surface free energy of polymers, *Journal of Applied Polymer Science* 13 (1969): 1741, <https://doi.org/10.1002/app.1969.070130815>.

[14]	F. Neese, The ORCA program system, *WIREs Computational Molecular Science* 2 (2011): 73, <https://doi.org/10.1002/wcms.81>.

[15]	W. Humphrey, A. Dalke, K. Schulten, VMD: visual molecular dynamics, *J Mol Graph* 14 (1996): 33, <https://doi.org/10.1016/0263-7855(96)00018-5>.

[16]	T. Lu, F. Chen, Multiwfn: a multifunctional wavefunction analyzer, *Journal of computational chemistry* 33 (2012): 580, <https://doi.org/10.1002/jcc.22885>.

[17]	A. Rose, Space-Charge-Limited Currents in Solids, *Physical Review* 97 (1955): 1538, <https://doi.org/10.1103/PhysRev.97.1538>.

[18]	C. A. Schneider, W. S. Rasband, K. W. Eliceiri, NIH Image to ImageJ: 25 years of image analysis, *Nat Methods* 9 (2012): 671, <https://doi.org/10.1038/nmeth.2089>.
